# Probing resonating valence bond states in artificial quantum magnets

**DOI:** 10.1038/s41467-021-21274-5

**Published:** 2021-02-12

**Authors:** Kai Yang, Soo-Hyon Phark, Yujeong Bae, Taner Esat, Philip Willke, Arzhang Ardavan, Andreas J. Heinrich, Christopher P. Lutz

**Affiliations:** 1 IBM Almaden Research Center, San Jose, CA USA; 2 Beijing National Laboratory for Condensed Matter Physics and Institute of Physics, Chinese Academy of Sciences, Beijing, China; 3 Center for Quantum Nanoscience, Institute for Basic Science (IBS), Seoul, Republic of Korea; 4 Department of Physics, Ewha Womans University, Seoul, Republic of Korea; 5 Ewha Womans University, Seoul, Republic of Korea; 6 CAESR, Clarendon Laboratory, Department of Physics, University of Oxford, Oxford, UK

**Keywords:** Scanning probe microscopy, Magnetic properties and materials

## Abstract

Designing and characterizing the many-body behaviors of quantum materials represents a prominent challenge for understanding strongly correlated physics and quantum information processing. We constructed artificial quantum magnets on a surface by using spin-1/2 atoms in a scanning tunneling microscope (STM). These coupled spins feature strong quantum fluctuations due to antiferromagnetic exchange interactions between neighboring atoms. To characterize the resulting collective magnetic states and their energy levels, we performed electron spin resonance on individual atoms within each quantum magnet. This gives atomic-scale access to properties of the exotic quantum many-body states, such as a finite-size realization of a resonating valence bond state. The tunable atomic-scale magnetic field from the STM tip allows us to further characterize and engineer the quantum states. These results open a new avenue to designing and exploring quantum magnets at the atomic scale for applications in spintronics and quantum simulations.

## Abstract (web summary)

The resonating valence bond state is a spin-liquid state where spins continuously alter their singlet partners. Here Yang et al. use spin-1/2 atoms precision-placed by a scanning tunnelling microscope to create artificial quantum magnets exhibiting the resonating valence bond state.

## Introduction

Antiferromagnetism is classically described by a Néel state with alternating spin orientations on neighboring atoms. However, in low-dimensional and low-spin antiferromagnets, quantum spin fluctuations are enhanced and tend to suppress the classical Néel order. This gives rise to exotic ground states lacking long-range magnetic order, such as quantum spin liquids^1^. A class of spin liquids is characterized by the resonating valence bond (RVB) state^2^, in which the quantum spins continuously alter their singlet partners. RVB states have been a central topic in quantum magnetism^3–5^ because they are believed to describe spin-1/2 Mott insulators such as the undoped copper oxides in high-*T*_c_ superconductors^6^. The RVB-type spin liquid has been observed by probing the spinon excitations using neutron scattering^7–9^ and nuclear magnetic resonance^10^. The low-energy excitations from the RVB states are fractional quasiparticles such as spinons and holons that enable spin-charge separation^11,12^ and result in low-loss spin transmission through insulators^11^.

RVB states in finite-sized spin arrays exhibit several important spin liquid properties such as a singlet ground state lacking conventional magnetic order, strong entanglement, and a stabilizing energy benefit from coherent RVB fluctuations^4,5^. Experimentally, these finite-size RVB states have been created in ensembles of ultracold atoms in optical lattices^5^, and in entangled photons^4^, providing new insights into the quantum correlations. However, RVB states with single-spin addressability have not yet been realized in a solid-state environment.

**Fig. 1:**
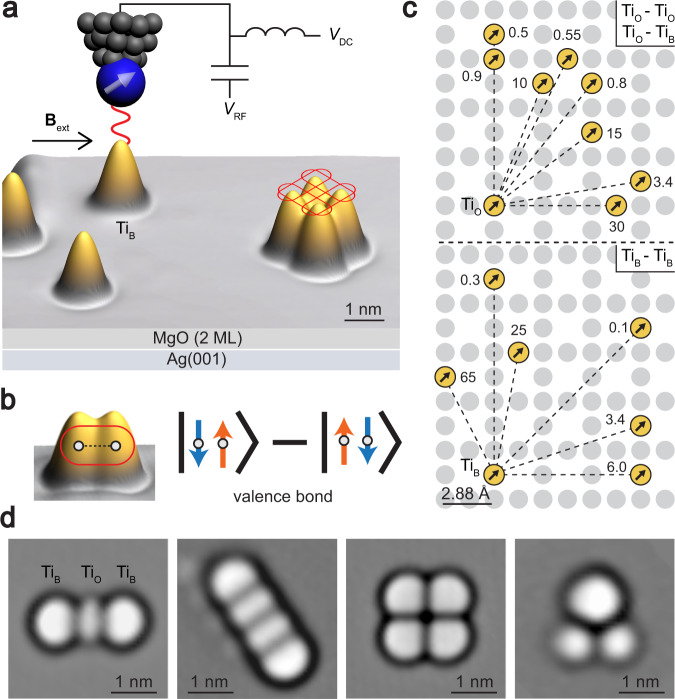
Toolbox of spin-1/2 atoms on a surface. **a** Experimental setup showing an STM with ESR capability, and an STM image (10 × 7.8 nm) of Ti atoms on MgO/Ag(001) (*V*_DC_ = 50 mV, *I*_DC_ = 30 pA). A spin-polarized tip is used to drive and sense ESR. Rounded rectangles represent valence bonds between Ti atoms positioned on the surface. **b** Valence bond (spin-singlet state), formed by two antiferromagnetically coupled Ti atoms. Blue and orange arrows represent spin-down and spin-up states, respectively. **c** Pairwise magnetic interactions (in units of GHz) for a range of different interatomic distances (Supplementary Table 1). Upper panel: Ti_O_–Ti_O_ and Ti_O_–Ti_B_ dimers. Lower panel: Ti_B_–Ti_B_ dimers. **d** Laplace-filtered STM images (3.5 × 3.5 nm) of different quantum magnets.

Atomic-scale magnetic structures such as spin chains and arrays have been constructed on surfaces using large-spin atoms in a scanning tunneling microscope (STM)^13–16^. Recently, the high energy resolution of magnetic resonance was combined with STM to coherently probe the states of individual spins^17^ and spin pairs on a surface^18–22^. Here, we used STM to perform electron spin resonance (ESR) of designed quantum magnets, in which we use spin-1/2 atoms to obtain strong quantum fluctuations, and we measured their many-body states with atomic resolution (Fig. 1a). This allows for the exploration of magnetic behaviors such as RVB states, which exhibit different degrees of quantum fluctuation and different excitation properties depending on their geometry. These quantum magnets provide a versatile quantum matter toolkit with atom-selective spin resonance spectroscopy that allows highly entangled states to be generated and probed in detail.

## Results

### Spin Hamiltonian of quantum magnets

Each quantum magnet was made by positioning Ti atomic spins on an insulating MgO film. Each Ti atom is hydrogenated, making it part of a TiH molecule^20,23^ and for simplicity, we refer to it simply as a Ti atom. Each Ti atom was adsorbed either on top of an oxygen atom (Ti_O_) or at a bridge site (Ti_B_) between two oxygen atoms^20,21^. At both sites it has spin *S* = 1/2^20,21^, giving it negligible magnetocrystalline anisotropy^24^ that could suppress spin fluctuations. Due to the antiferromagnetic exchange interaction at close distance^20,21^, a pair of Ti spins forms a singlet ground state $\circ \hskip -4pt-\hskip -4pt \circ = {\kern 1pt} \downarrow \uparrow - \uparrow \downarrow$ (normalization factors omitted throughout) (Fig. 1b), known as a valence bond^5,6^. Here ↑ and ↓ represent spin-up and spin-down states, respectively. In structures containing more than two spins, quantum fluctuations between many such valence bonds can give rise to the RVB state, in which the spins continuously alter their singlet partners and rearrange the pairings.

To design a quantum magnet exhibiting RVB states, we first characterized the pairwise magnetic interaction *J* for different Ti pairs by measuring the splitting of ESR peaks as a function of distance^20^, as shown in Fig. 1c. We used the Ti spins coupled dominantly by antiferromagnetic exchange (*J*_*ij*_ > 0) to build quantum magnets, including odd- and even-length spin chains, spin triangles, and spin plaquettes (Fig. 1d and Supplementary Fig. 1). The quantum states of these quantum magnets under external applied magnetic field **B**_ext_ (in-plane) are described by the Hamiltonian^20,25^:

1 $$H = \mathop {\sum}\limits_{i,j} {J_{ij}{\mathbf{S}}_i \cdot {\mathbf{S}}_j + } \mathop {\sum}\limits_i {g\mu _{\mathrm{B}}{\mathbf{B}}_{{\mathrm{ext}}} \cdot {\mathbf{S}}_i + g\mu _{\mathrm{B}}{\mathbf{B}}_{{\mathrm{tip}}} \cdot {\mathbf{S}}_n}$$

where **S**_*i*_ is the spin operator for site *i*. The g-factor *g* ≈ 1.8 is obtained by ESR of isolated Ti atoms^20,25^, and *μ*_B_ is the Bohr magneton. The antiferromagnetic interactions compete with the Zeeman term, which tends to align the spins along **B**_ext_. Here, we used an isotropic spin-1/2 model as an approximation which agrees well with most of the experimental data and thus captures the main physics. Consideration of g-factor anisotropy^23^ could further improve the accuracy of the spin Hamiltonian. Note that **B**_ext_ is the only source of anisotropy in the Hamiltonian, so changing its direction (here it is applied in-plane) should not affect the form of the eigenstates or energies. The atomic-scale tip magnetic field **B**_tip_ (Supplementary Fig. 2) is used both to drive ESR transitions and to tune the spin states of the quantum magnets by exerting an exchange bias only on the spin **S**_*n*_ under the tip^25^. Here, the ESR transitions between two coupled-spin states $\left| i \right\rangle$ and $\left| j \right\rangle$ are allowed if there is a nonzero matrix element $\left\langle i \right|{\Delta} {\mathbf{B}}_{{\mathrm{tip}}} \cdot {\mathbf{S}}_n\left| j \right\rangle$, where Δ**B**_tip_ gives the field gradient^20^. We are thus able to drive ESR transitions between different spin multiplets, which are forbidden in traditional spin resonance^26^, offering direct access to the energy differences between multiplets.

### RVB states in an odd number chain

**Fig. 2:**
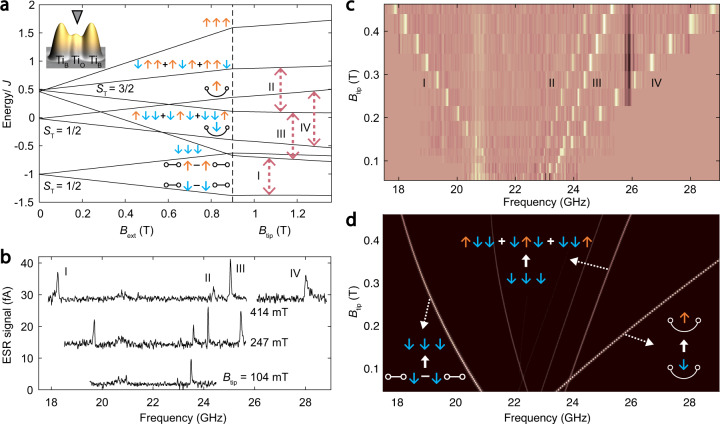
Magnetic states of a three-spin chain. **a** Energy level diagram as a function of *B*_ext_ (up to 0.9 T), and *B*_tip_ with *J* ≈ 30 GHz, calculated using Hamiltonian (1). The scale shows the sum of *B*_ext_ and *B*_tip_. States are labeled by their wavefunctions at *B*_tip_ = 0. Blue and orange arrows represent spin-down and spin-up states, respectively (same below). Dashed arrows indicate detected ESR transitions. **b**, **c** ESR spectra measured on the middle (Ti_O_) spin at different *B*_tip_ as labeled, shown as a graph (**b**) and lightness scale (**c**). (*V*_DC_ = 60 mV, *I*_DC_ = 5–40 pA, *V*_RF_ = 7–22 mV, *B*_ext_ = 0.9 T). Signals near 20.6 and 25.9 GHz are experimental artifacts due to the frequency-dependent RF transmission. **d**, Simulated ESR spectra. Weak anisotropy^48^ (−0.02 *J*) is added to the exchange Hamiltonian to reproduce the observed slightly broken degeneracy, yielding distinct peaks II and III (Supplementary Note 5).

We first built a three-spin chain by alternating Ti_O_ and Ti_B_ atoms to obtain the nearest-neighbor coupling of *J* ≈ 30 GHz (Fig. 2). This coupling strength results in a low-spin ground state, while allowing transitions between different multiplets to be visible in our ESR range of ~10–30 GHz. Each spin multiplet with a total spin *S*_T_ fans out into its 2*S*_T_ + 1 components, each having a different quantum number *M*, when **B**_ext_ is increased (Fig. 2a and Supplementary Fig. 3). The three-spin chain has a ground state $\left| 1 \right\rangle = 2\left( { \downarrow \uparrow \downarrow } \right) - \left( { \downarrow \downarrow \uparrow + \uparrow \downarrow \downarrow } \right) = \circ \hskip -4pt-\hskip -4pt \circ \downarrow - \downarrow \circ \hskip -4pt-\hskip -4pt \circ$. This coherent superposition of valence bond states is a resonance between $\circ \hskip -4pt-\hskip -4pt \circ \downarrow$ and $\downarrow \circ \hskip -4pt-\hskip -4pt \circ$, giving an energy of −*J*, which is lower than the energy expectation for states having only one valence bond ($\circ \hskip -4pt-\hskip -4pt \circ \downarrow$ or $\downarrow \circ \hskip -4pt-\hskip -4pt \circ$) or for a Néel state (↓↑↓). The case of a single valence-bond state would arise in the limiting case where $J_{12} \gg J_{23}$. The extreme case is the third spin being isolated from the other two spins, and evolution of the ESR spectra measured on the middle spin would be the same as those measured on a spin dimer^21^. In contrast, Néel states should appear only for spins having large enough magnetic anisotropy^27^ or in arrays having sufficient disorder to mix the RVB state with higher-lying states. This 3-spin chain represents the simplest example of an RVB state, illustrating how a coherent superposition of valence bond states gives an energy benefit.

To probe the energy spectrum and eigenstates of this three-spin chain, we performed ESR on the middle spin and studied the evolution of the ESR spectra as a function of *B*_tip_ (Fig. 2b, c). Since different ESR transitions respond differently with increasing *B*_tip_, this allows us to easily identify the corresponding initial and final states, in accordance with the quantitative simulations (Fig. 2d).

For example, the lowest-frequency ESR peak (I) reveals the transition from the ground state $\left| 1 \right\rangle = \circ \hskip -4pt-\hskip -4pt \circ \downarrow - \downarrow \circ \hskip -4pt-\hskip -4pt \circ$ (*S*_T_ = 1/2, *M* = −1/2), to the ferromagnetic state $\left| 2 \right\rangle = \downarrow \downarrow \downarrow$ (*S*_T_ = 3/2, *M* = −3/2). The average spin polarization $\langle S_2^z\rangle$ (where *z* is the direction of **B**_ext_) of the middle spin is zero in the state $\left| 1 \right\rangle$, while in state $\left| 2 \right\rangle$ it is fully polarized. This difference in local spin polarization is directly visualized as the frequency shift of peak I with increasing *B*_tip_, which favors the spin polarization of the middle spin in state $\left| 2 \right\rangle$. For the higher-energy states $\left| 4 \right\rangle = {\,}$ and $\left| 6 \right\rangle = {\,}$, the two end spins form a spin-singlet. These two states are also eigenstates of the spin Hamiltonian having zero energies at zero magnetic field since the direct coupling between the two end spins is negligible. The polarization of the center spin is opposite for these two states, so the corresponding ESR transition (IV) shifts to higher frequency rapidly as we increase *B*_tip_.

The observation of the ESR transitions I and III allows us to determine the energy of the RVB ground state with respect to the ferromagnetic state, which is a conventional measure of RVB energy^28^ that gives the quantitative energy benefit for forming this coherent superposition of singlets. Here, it is given by $- \left( {f_{\mathrm{I}} + f_{{\mathrm{III}}}} \right)/3 = \left( { - 0.497 \pm 0.010} \right){J}$ per spin at zero magnetic field (Supplementary Fig. 5), in agreement with the calculated value of −0.5 *J*.

### RVB states in an even number chain

**Fig. 3:**
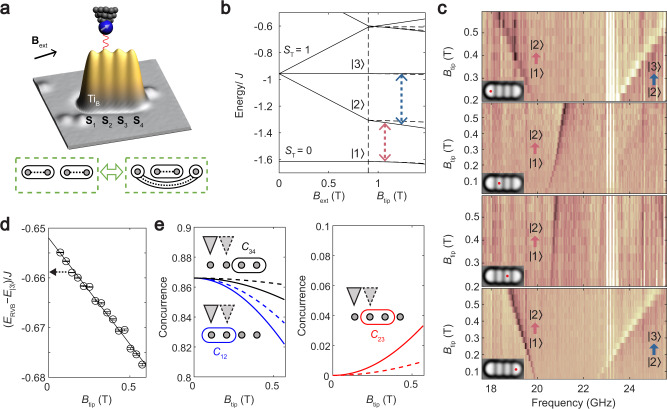
Spin pairings in an even-length chain. **a** Experimental setup showing a four-spin chain of Ti_B_ atoms (*J* ≈ 65 GHz). The double arrow indicates the superposition of two spin-pairing configurations of the ground state $\left| 1 \right\rangle$. **b** Energy level diagram as a function of *B*_ext_ and *B*_tip_, applied to **S**_1_ (solid lines) or **S**_2_ (dashed lines). Dashed arrows indicate detected ESR transitions. **c** ESR spectra measured on each of the 4 spins as a function of *B*_tip_ (*V*_DC_ = 60 mV, *I*_DC_ = 10–160 pA, *V*_RF_ = 24–38 mV, *B*_ext_ = 0.9 T). Vertical stripes are due to the frequency-dependent RF transmission. **d** Energy difference between states $\left| 1 \right\rangle$ and $\left| 3 \right\rangle$ at different *B*_tip_, with a linear fit (solid line). Dashed arrow indicates the theoretical value at *B*_tip_ = 0. Error bars are from the fitting uncertainties of the ESR frequencies with a 95% confidence. **e** Ground-state concurrence *C*_*ij*_ between the three different pairs of neighboring spins, as a function of *B*_tip_. Solid (dotted) lines show concurrence when the *B*_tip_ is applied to **S**_1_ (**S**_2_) as illustrated.

Quantum magnets having an even number of spins^14^ can exhibit a non-magnetic RVB ground state because all the spins can simultaneously pair into valence bonds. Resonance among different ways to pair the spins results in intriguing spin entanglement between pairs that are not directly coupled^4,29,30^. To explore these properties, we built a four-spin chain with a strong pairwise coupling of ~65 GHz (Fig. 3a), which exceeds the Zeeman energy of individual spins at 0.9 T so that the RVB state, which is non-magnetic (*S*_T_ = 0), becomes the ground state (Fig. 3b and Supplementary Fig. 8). The RVB state is the superposition of two pairing configurations: $\left( { \downarrow _1 \uparrow _2 - \uparrow _1 \downarrow _2} \right)\left( { \downarrow _3 \uparrow _4 - \uparrow _3 \downarrow _4} \right)$ and $\left( { \downarrow _1 \uparrow _4 - \uparrow _1 \downarrow _4} \right)\left( { \downarrow _2 \uparrow _3 - \uparrow _2 \downarrow _3} \right)$ (Fig. 3a, bottom). Even though there is negligible direct pairwise interaction between the end spins **S**_1_ and **S**_4_, the singlet bonding configuration for these spins still appears in the ground state. Thus, the RVB state can be envisaged as a fluctuating pattern of valence bonds having different lengths^2^. We measured the ESR spectra on different sites as a function of *B*_tip_ (Fig. 3c). This allowed us to extract information about the spin wavefunction, including spin polarization on different sites and spin pairings. Conventional tunneling spectra (d*I*/d*V*) are thermally limited so they resolve only a single broadened excitation for this chain (Supplementary Fig. 6).

The ESR transition from the RVB ground state $\left| 1 \right\rangle$ to the first excited state $\left| 2 \right\rangle$ (*S*_T_ = 1, *M* = −1) evolves differently depending on where $B_{{\mathrm{tip}}}$ is applied. Since all spins have zero polarization in state $\left| 1 \right\rangle$, this evolution gives us information about the spin distributions of the state $\left| 2 \right\rangle$ with atomic resolution. Examination of the quantitative spin states shows that for state $\left| 2 \right\rangle$ the *z*-axis spin polarization of **S**_1_ (~85%) is larger than **S**_2_ (~15%) (Supplementary Fig. 7). Accordingly, the frequency of the ESR transition from the state $\left| 1 \right\rangle$ to $\left| 2 \right\rangle$ shifts faster when $B_{{\mathrm{tip}}}$ is applied to **S**_1_ than the case with *B*_tip_ applied on **S**_2_ (Fig. 3c). At higher frequency, the transition from $\left| 2 \right\rangle$ to $\left| 3 \right\rangle$ is visible when measuring the spin at either end of the chain.

This atomic-scale ESR offers a direct measurement of the energy differences between spin multiplets, due to its distinct selection rule based on atomically local excitation, while these relative energies are not accessible in conventional bulk ESR^26^. For example, the sum of the frequencies of the two detected ESR transitions gives the energy difference between the two lowest spin multiplets (Fig. 3d), which is $( - 0.652 \pm 0.010)\,{J}$ at zero field, in agreement with the calculated value of −0.659 *J*.

The atomic-scale tip field also influences the pairwise entanglement in the RVB ground state through its competition with the exchange coupling and the external field. To qualitatively understand this competition, we plot the concurrence *C*_*ij*_, a measure of entanglement^4,29,30^, between two spin sites *i* and *j* computed for the ground state (Fig. 3e and Supplementary Note 4). The concurrences varied differently depending on whether *B*_tip_ was applied on **S**_1_ or **S**_2_. This sensitivity directly reflects the spin-pairing information. The tip field only has to compete with $J{\mathbf{S}}_1 \cdot {\mathbf{S}}_2$ to polarize spin 1, while it has to compete with both $J{\mathbf{S}}_1 \cdot {\mathbf{S}}_2$ and $J{\mathbf{S}}_2 \cdot {\mathbf{S}}_3$ when *B*_tip_ is applied on spin 2. Note that the decrease of *C*_12_ is accompanied by the increase of *C*_23_, showing the “monogamy” of entanglement^4,29^.

### RVB states in a spin plaquette

**Fig. 4:**
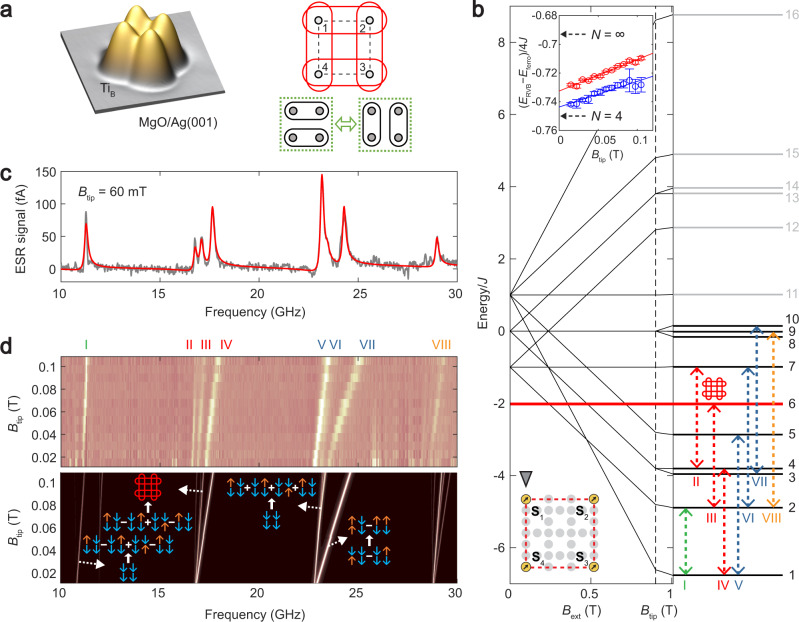
RVB state in a spin plaquette. **a** STM image of a spin plaquette of Ti_B_ atoms (*J* ≈ 6 GHz). Right: Schematic of the RVB state (red) consisting of the superposition of two spin pairings (green). **b** Energy level diagram as a function of *B*_ext_ and *B*_tip_. Dashed arrows indicate detected ESR transitions. Red line is the RVB state energy. Upper inset: energy per spin of the RVB state relative to the ferromagnetic state $\downarrow _1 \downarrow _2 \downarrow _3 \downarrow _4$ at *B*_ext_ = 0, determined in two ways, by measuring $\left( {f_{\mathrm{I}} + f_{{\mathrm{III}}} - 2g\mu _{\mathrm{B}}B_{{\mathrm{ext}}}} \right)/4{J}$ (red circles) or $\left( {f_{{\mathrm{II}}} + f_{{\mathrm{III}}} + f_{{\mathrm{IV}}} - f_{{\mathrm{VI}}} - 2g\mu _{\mathrm{B}}B_{{\mathrm{ext}}}} \right)/4{J}$ (blue circles) and extrapolating to $B_{{\mathrm{tip}}} = 0$. Solid lines are linear fits. Dashed arrows indicate the theoretical values for *N* = 4 spins and *N* = ∞^28^ at $B_{{\mathrm{tip}}} = 0$. Error bars are from the fitting uncertainties of the ESR frequencies with a 95% confidence. **c** ESR spectra taken on **S**_1_ (*V*_DC_ = 50 mV, *I*_DC_ = 200 pA, *V*_RF_ = 8 mV, *B*_ext_ = 0.9 T). Red curve is a fit to a sum of asymmetric Lorentzian peaks. **d** ESR spectra as a function of *B*_tip_ of the spin plaquette (*V*_DC_ = 50 mV, *I*_DC_ = 50–350 pA, *V*_RF_ = 3–13 mV, *B*_ext_ = 0.9 T). Lower panel: Simulated ESR spectra. Blue and orange arrows represent spin-down and spin-up states, respectively.

While the four-spin chain shows an unequal superposition of the valence bond basis states, we can engineer an equal superposition by building closed-chain structures, with additional translational symmetry. A closed chain of four spins is obtained by assembling a 2-by-2 plaquette^31^ (Fig. 4a), the smallest configuration for simulating a quantum spin liquid on a two-dimensional square lattice^3–5^. In such a geometry, there are two valence-bond basis states (Supplementary Figs. 10, 11): $| {{\mathrm{{\Phi} }}_ = } \rangle = \left( { \downarrow _1 \uparrow _2 - \uparrow _1 \downarrow _2} \right)\left( { \downarrow _4 \uparrow _3 - \uparrow _4 \downarrow _3} \right)$ and $| {{\mathrm{{\Phi} }}_\parallel } \rangle = \left( { \downarrow _1 \uparrow _4 - \uparrow _1 \downarrow _4} \right)\left( { \downarrow _2 \uparrow _3 - \uparrow _2 \downarrow _3} \right)$. The RVB state has *s*-wave symmetry and consists of the coherent superposition $| {{\mathrm{{\Phi} }}_ = } \rangle + | {{\mathrm{{\Phi} }}_\parallel } \rangle$, resulting in the entanglement of each spin with its two neighbors (Fig. 4a, right). The *d*-wave superposition $| {{\mathrm{{\Phi} }}_ \times } \rangle = | {{\mathrm{{\Phi} }}_ = } \rangle - | {{\mathrm{{\Phi} }}_\parallel } \rangle$, in contrast, is not an RVB state and lies at higher energy (Supplementary Fig. 10).

We constructed a plaquette with an interatomic distance *d* = 8.7Å (Fig. 4a), yielding a nearest-neighbor coupling $J \approx 6\,{\mathrm{GHz}}$, and negligible second-nearest-neighbor coupling (∼0.1 GHz). At *B*_ext_ = 0.9 T, the Zeeman energy dominates, and therefore the ferromagnetic state $\downarrow _1 \downarrow _2 \downarrow _3 \downarrow _4$ is the ground state (Fig. 4b). Many transitions are accessible to ESR in such a plaquette because of the thermal occupation of several lower-lying states (Fig. 4c, d). These transitions are in excellent agreement to the energies, amplitudes, and the dependence on *B*_tip_ given by diagonalization of the model Hamiltonian (quantum states detailed in Supplementary Fig. 11).

We obtain direct access to the RVB state by driving an ESR transition (peak III in Fig. 4) into the RVB state. We can thus measure the relative energy between the RVB state and the ferromagnetic state ($\downarrow _1 \downarrow _2 \downarrow _3 \downarrow _4$), giving $( - 0.738 \pm 0.030)\,{J}$ per spin (Fig. 4b, upper inset). This value agrees with the expected value of −0.75 *J* for an ideal spin-1/2 plaquette, and is much lower than the −0.5 *J* of a Néel state ($\downarrow _1 \uparrow _2 \downarrow _3 \uparrow _4$), thus confirming the RVB nature of this spin plaquette. Theoretical studies show that the energy per spin decreases in magnitude with an increasing number of spins, and reaches −0.693 *J* in the thermodynamic limit of an infinite chain^28^, which is remarkably similar to the four-spin value. We further brought four Ti atoms closer to form a smaller plaquette $(d = 7.4\,{\mathrm{{\AA}}})$, which increased the coupling *J* to 25 GHz, making the RVB state lowest in energy even in the presence of the magnetic field (Supplementary Note 8).

## Discussion

The formation of the RVB state studied here is due to the competition between different configurations of spin-singlet pairings. This competition exists even though the Heisenberg antiferromagnet on a square lattice does not have classical magnetic frustration, which requires odd-length cycles^2^. We also generated such classical frustration, coexisting with RVB behavior, by assembling Ti atoms into a spin triangle (Supplementary Note 9), and observe that the frustration brings the two low-spin doublets closer in energy than in the 3-spin chain, leading towards the high-degeneracy ground state characteristic of frustrated systems. Geometrically frustrated lattices with competing interactions are highly challenging for analytical and numerical studies. Employing the atomic-scale ESR technique developed here for probing coupled-spin states, and using a different decoupling layer such as hexagonal boron nitride, one could measure the emergent non-trivial phases of the spin-1/2 triangular lattice.

Custom-designed quantum magnets assembled on a surface combined with single-atom ESR provide a flexible platform to explore the quantum states of finite-size spin systems. This technique can introduce precisely characterized disorder by placing point defects, vacancies, and adjusted couplings by repositioning the atoms. These artificial nanomagnets could aid in the design and complement the use of chemically synthesized molecular nanomagnets^32–34^, which have emerged as promising vehicles for spintronics^35^, quantum computing^34,36,37^, and quantum simulations^38^. The precisely engineered finite-size quantum many-body systems demonstrated here may serve as versatile analog quantum simulators^31,34,38,39^ because they can be assembled, modified, and probed in situ with single-spin selectivity. The spin plaquette constructed here is the fundamental building block of the square lattice spin liquids^40^. A unique opportunity provided by the STM is to explore the real-space response of the spin liquid to point defects such as individual pinned magnetic moments, which can better reveal the character of the quantum spin liquids^41,42^. In addition, studies of larger spin arrays or using different atom species with larger single-ion anisotropy should allow exploration of the quantum-classical-transition, and of competition between quantum fluctuations and Néel order^8,43^. Another natural extension of the current work is to use atomic spins on MgO to realize simulations of the magnetic phases of the Mott insulating states in copper oxide high-*T*_c_ superconductors, and this could also provide a realization of the deconfined quantum critical point by tuning the quantum magnets^44^.

In addition to exhibiting stationary magnetic orderings, quantum spin arrays can also carry spinon spin current based on quantum fluctuations^11^. The combination of pump–probe electronic pulses^45^ with pulsed ESR^22^ could allow further exploration of the quantum dynamics of quasiparticles in artificial spin structures with atomic resolution, including, for example, the dynamical evolution of the spin transmission in spin arrays on surfaces, or the operation of nanoscale devices based on spin currents in insulators^11,46^. Studying the time evolution and confinement of these elementary excitations in 1D and 2D could help to reveal how information propagates in many-body systems, complementing numerical simulations and analytical studies^47^.

## Methods

### Sample preparation

Measurements were performed in a home-built ultrahigh-vacuum (<10^−9^ Torr) STM operating at 1.2 K. MgO is two monolayers (ML) thick (referred to as bilayer MgO) and was grown on an atomically clean Ag(001) single crystal by thermally evaporating Mg in an ~10^–6^ Torr O_2_ environment^17^. Ti and Fe atoms were deposited in situ from pure metal rods by e-beam evaporation onto the sample held at ~10 K. An external magnetic field (0.44 T, 0.5 T or 0.90 T as indicated in the figure captions) was applied at ~8° off the surface, with the in-plane component aligned along the [100] direction of the MgO lattice. STM images were acquired in constant-current mode and all voltages refer to the sample voltage with respect to the tip.

### Spin-polarized tip

The iridium STM tip was coated with silver by indentations into the Ag sample until the tip gave a good lateral resolution in the STM image. To prepare a spin-polarized tip, ~1–5 Fe atoms were each transferred from the MgO onto the tip by applying a bias voltage (~0.55 V) while withdrawing the tip from near point contact with the Fe atom. The degree of spin polarization was verified by the asymmetry in d*I*/d*V* spectra of Ti with respect to voltage polarity^20^.

### RF measurement

The continuous wave electron spin resonance spectra were acquired by sweeping the frequency of an RF voltage *V*_RF_ generated by the RF generator (Agilent E8257D) across the tunneling junction and monitoring changes in the tunneling current. The current signal was modulated at 95 Hz by chopping *V*_RF_, which allowed the readout of the current by a lock-in technique^17^. The RF and DC voltages were combined at room temperature using an RF diplexer, and guided to the STM tip through semi-rigid coaxial cables with a loss of ~30 dB at 20 GHz^17^.

## Supplementary information

Supplementary Information

Supplementary Movie 1

Supplementary Movie 2

Description of Additional Supplementary Files

## Data Availability

The data that support the plots within this paper and other findings of this study are available from the corresponding authors upon reasonable request.
