# Supplementary material for: Probing resonating valence bond states in artificial quantum magnets
Source: Nat Commun. 2021 Feb 12;12:993. doi: 10.1038/s41467-021-21274-5 (PMC7881118; doi:10.1038/s41467-021-21274-5)
Supplement: Supplementary file 1 — Supplementary Information [file 41467_2021_21274_MOESM1_ESM.pdf]

Supplementary Information

Probing resonating valence-bond states in artificial quantum magnets

Kai Yang,[1,2,*] Soo-Hyon Phark,[1,3,4] Yujeong Bae,[1,3,5] Taner Esat,[1,3,5] Philip Willke,[1,3,5] Arzhang Ardavan,[6] Andreas J. Heinrich,[3,4,*] and Christopher P. Lutz[1,*]

[1]IBM Almaden Research Center, San Jose, CA 95120, USA

[2]Beijing National Laboratory for Condensed Matter Physics and Institute of Physics, Chinese Academy of Sciences, Beijing 100190, China

[3]Center for Quantum Nanoscience, Institute for Basic Science (IBS), Seoul 03760, Republic of Korea

[4]Department of Physics, Ewha Womans University, Seoul 03760, Republic of Korea

[5]Ewha Womans University, Seoul 03760, Republic of Korea

[6]CAESR, Clarendon Laboratory, Department of Physics, University of Oxford, Oxford OX1 3PU, UK.

*Corresponding authors: K.Y. (kaiyang@iphy.ac.cn), A.J.H. (heinrich.andreas@qns.science) and C.P.L. (cplutz@us.ibm.com)

Table of contents

Supplementary Note 1. Building quantum magnets by STM manipulations 2
Supplementary Note 2. Characterizing the tip magnetic field 3
Supplementary Note 3. Modeling of the ESR spectra 4
Supplementary Note 4. Calculations of the concurrence 4
Supplementary Note 5. 3-spin chain (J = 30 GHz) 5
Supplementary Note 6. 4-spin chain (J = 65 GHz) 7
Supplementary Note 7. Spin plaquette (J = 6 GHz) 11
Supplementary Note 8. Spin plaquette (J = 25 GHz) 13
Supplementary Note 9. Spin triangle 15
Supplementary Note 10. References 18

Supplementary Note 1. Building quantum magnets by STM manipulations

The quantum magnets were built by lateral manipulation with the STM tip using typical junction resistance of 50 MΩ (Supplementary Fig. 1). See also Supplementary Movies 1 and 2 for how the spin plaquette and chain were built. For long-distance movement (> 1 nm), we dragged the Ti atoms using the STM tip with the constant-current feedback loop closed (V_{DC} = 0.35 V, I_{DC} = 7 nA). For the precise atomic manipulation, we first positioned the STM tip above the destination binding site at a typical junction resistance of 50 MΩ, and then ramped the V_{DC} from 1 mV to ~0.3 V to attract the Ti atom with the feedback loop open.

(a)

(b)

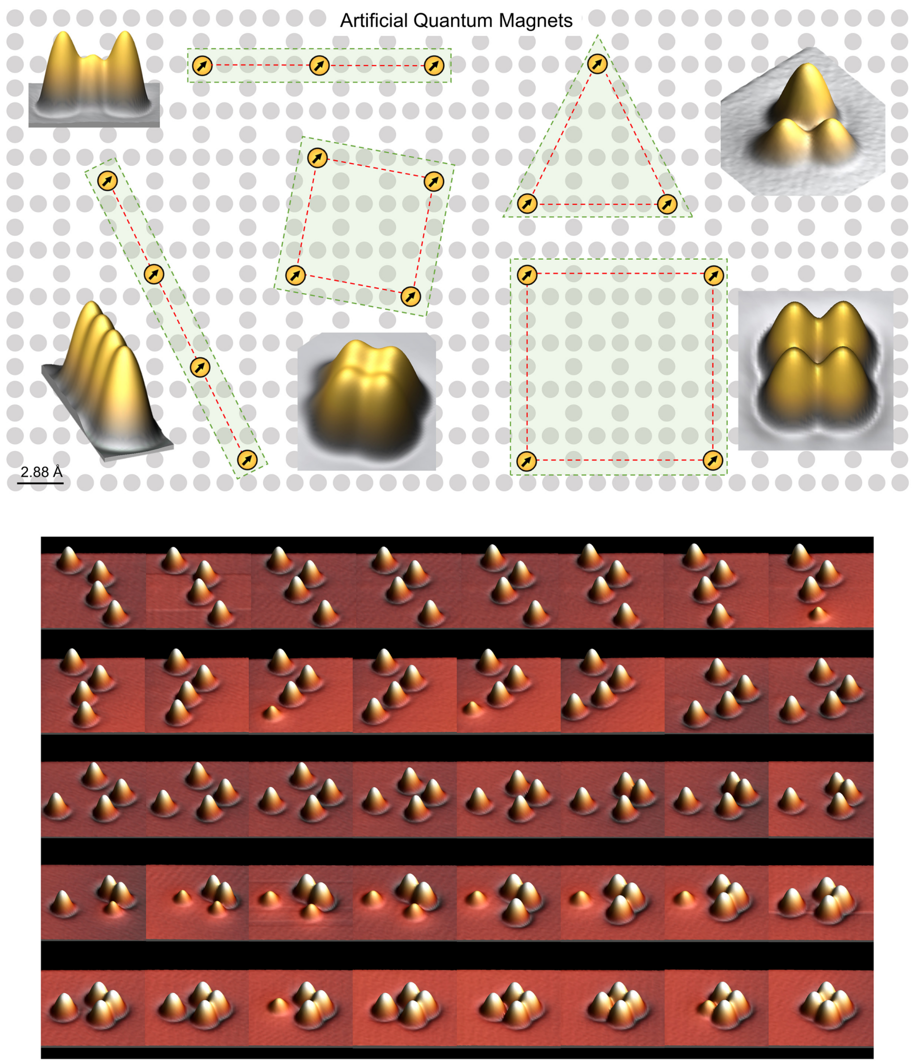

Supplementary Fig. 1. (a) Artificial quantum magnets built by Ti atoms on 2 monolayers (ML) MgO/Ag(001). The lattice constant of MgO is 2.88 Å. (b) A series of STM images (6 nm × 6 nm or 5 nm × 5 nm) showing the process of building a spin plaquette (8.6 Å spacing, J = 6 GHz) using Ti atoms on MgO/Ag(001) (V_{DC} = 0.1 V, I_{DC} = 10 pA). The Ti_O atoms appear smaller in the topographic height than the Ti_B atoms.

The exchange coupling constant J shows exponential dependence on the separation r between Ti atoms[1,2]. From the ESR spectra, we find the exchange coupling constant J can be described by an exponential function, $J = J_0 \exp\left(-(r - r_0)/d\right)$ where r_0 is an arbitrary reference distance.

Type of dimers	Coupling prefactor J_0	Decay constant d	r_0
Ti_O-Ti_B	28.9 ± 1.3 GHz	0.65 ± 0.05 Å	7.2 Å
Ti_B-Ti_B	27.7 ± 0.1 GHz	0.94 ± 0.01 Å	7.2 Å

Supplementary Table 1. Exchange coupling constant J for Ti_O-Ti_B and Ti_B-Ti_B dimers[2].

Supplementary Note 2. Characterizing the tip magnetic field

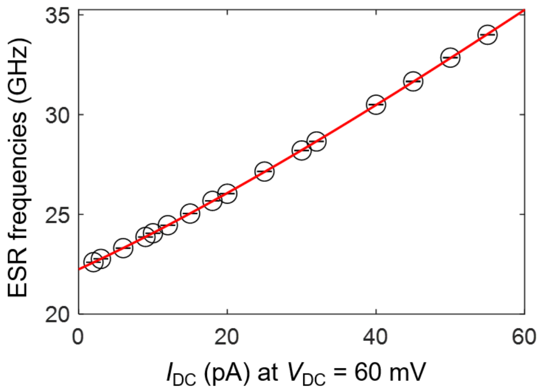

Supplementary Fig. 2. Fitted ESR frequencies of a single Ti_O atom at different tip-Ti distances set by different I_{DC} at V_{DC} = 60 mV (B_{ext} = 0.9 T, T = 1.2 K). Error bars are from the fitting uncertainties of the ESR frequencies with a 95% confidence.

The direction and amplitude of the tip magnetic field can be obtained by measuring the ESR spectra on a single Ti atom at different tip-Ti distances[3]. The Zeeman energy (E_{total}) of a single Ti atom includes the contributions from both $\mathbf{B}_{ext}$ and $\mathbf{B}_{tip}$:

$$E_{total}(z) = \sqrt{E_{ext}^2 + E_{tip}^2(z) + 2E_{ext}E_{tip}(z)\cos\theta} \quad \text{(S1)}$$

Here $E_{ext} = \gamma\hbar B_{ext}$, $E_{tip}(z) = \gamma\hbar B_{tip}(z) \propto \exp\left(-z/d_{ex}\right)$, and θ is the angle between $\mathbf{B}_{ext}$ and $\mathbf{B}_{tip}$. At constant V_{DC}, we use the approximation $E_{tip}(z) \propto I_{DC}$ since the decay lengths (~0.5 Å) of the exchange interaction and the tunnelling current are nearly equal. By fitting E_{total} as a function of tip-Ti distance using equation (S1) (Supplementary Fig. 2), we find $\theta \approx 52.9°$ and $B_{tip} \approx 11.5$ mT/pA for the STM tip used for measuring the data in Fig. 2. Similar measurement yields $\theta \approx 52.9°$ and $B_{tip} \approx 3.6$ mT/pA for the tip used in Fig. 3; $\theta \approx 0°$ and $B_{tip} \approx 0.3$ mT/pA for the tip used in Fig. 4.

Supplementary Note 3. Modeling of the ESR spectra

When the tip is positioned over the atom with a spin operator $\mathbf{S}_n$, the ESR transition k between two coupled-spin states $|i\rangle$ and $|j\rangle$ is allowed if there is a nonzero matrix element $\langle i|\Delta\mathbf{B}_{\text{tip}} \cdot \mathbf{S}_n|j\rangle$. Here $\Delta\mathbf{B}_{\text{tip}}$ is the magnetic field gradient of the tip magnetic field $\mathbf{B}_{\text{tip}}$, which is along the same direction as $\mathbf{B}_{\text{tip}}$ assuming an isotropic tip-Ti exchange interaction[3]. The ESR spectrum with N transitions is described by[1,2,4]:

$$I^{\text{ESR}} = I_0^{\text{ESR}} + \sum_k^N I_k^{\text{ESR}} \cdot \frac{1 + q \cdot \nu_k}{1 + \nu_k^2} \tag{S2}$$

Here the normalized RF frequency $\nu_k = (f_{\text{RF}} - f_k)/(\Gamma/2)$ and ESR linewidth $\Gamma = \frac{1}{\pi T_2}\sqrt{1 + \Omega_k^2 T_1 T_2}$. The Rabi rate of transition k is $\Omega_k \propto V_{\text{RF}}|\langle i|\Delta\mathbf{B}_{\text{tip}} \cdot \mathbf{S}_n|j\rangle| \propto V_{\text{RF}}|\langle i|\mathbf{B}_{\text{tip}} \cdot \mathbf{S}_n|j\rangle|$. q is the asymmetry factor of the line shape due to the homodyne detection of the ESR signal[2]. The measured ESR signal amplitude I_k^{ESR} of transition k is related to the thermal equilibrium populations p_i and p_j by[1,4]

$$I_k^{\text{ESR}} \propto \left|\mathbf{B}_{\text{tip}} \cdot (\langle \mathbf{S}_n \rangle_j - \langle \mathbf{S}_n \rangle_i)\right| \cdot \left|p_j - p_i\right| \frac{\Omega_k^2 T_1 T_2}{1 + \Omega_k^2 T_1 T_2} \tag{S3}$$

We used equation (S2) to fit the ESR spectra to obtain the ESR frequencies. For the ESR simulations, we set $q = 0$, and $\Gamma = 20$ MHz, and used a tip magnetic field measured as described in Supplementary Note 2.

Supplementary Note 4. Calculations of the concurrence

Concurrence gives a measure of entanglement of two spins that is entanglement-monotone and can be calculated readily. We calculated the concurrence following the procedure in Ref. 5, which is briefly described as follows. For a reduced density matrix ρ of two spins, the 2-spin density matrix $\tilde{\rho}$ is defined as $\tilde{\rho} = (\sigma_{1y} \otimes \sigma_{2y})\rho^*(\sigma_{1y} \otimes \sigma_{2y})$. Here σ_{1y} and σ_{2y} are the y Pauli matrices, and ρ^* is complex conjugate of ρ taken in the Zeeman product basis, which is $\{\uparrow\uparrow, \uparrow\downarrow, \downarrow\uparrow, \downarrow\downarrow\}$.

The concurrence C for a mixed state ρ of two spins is calculated by

$$C = \max\{\lambda_1 - \lambda_2 - \lambda_3 - \lambda_4, 0\} \tag{S4}$$

where λ_is are the square roots of the eigenvalues of the non-Hermitian matrix $\rho\tilde{\rho}$.

Supplementary Note 5. 3-spin chain (J = 30 GHz)

The spin Hamiltonian of the 3-spin chain is:

$$H = J(\mathbf{S}_1 \cdot \mathbf{S}_2 + \mathbf{S}_2 \cdot \mathbf{S}_3) + J_{\mathrm{ani}}[(S_1^z + S_2^z)^2 + (S_2^z + S_3^z)^2] + g\mu_{\mathrm{B}}\mathbf{B}_{\mathrm{ext}} \cdot (\mathbf{S}_1 + \mathbf{S}_2 + \mathbf{S}_3) + g\mu_{\mathrm{B}}\mathbf{B}_{\mathrm{tip}} \cdot \mathbf{S}_2 \quad \text{(S5)}$$

Here the term with a prefactor J_{ani} accounts for the anisotropy of the exchange interaction[6]. In Supplementary Fig. 3, we show the energy levels of the 3-spin chain with the exchange interaction anisotropy ($J_{\mathrm{ani}} = -0.02\,J$). By comparing Fig. 2c with Fig. 2d (reproduced as Supplementary Fig. 4b), we find that the weakly anisotropic exchange well reproduces the evolution of the ESR peaks, including the splitting of transitions II and III, of the 3-spin chain.

It is recently reported that the Ti_O atom on MgO has a g-factor anisotropy[7]. Including g-anisotropy in the simulation (Supplementary Fig. 4c, d) also results in the splitting of transitions II and III, but the splitting increases with increasing B_{tip}, different from the measured constant splitting at different B_{tip} (Fig. 2c). This suggests that the g-factor anisotropy plays a less important role than the anisotropy of the exchange interaction for the 3-spin chain.

(a)

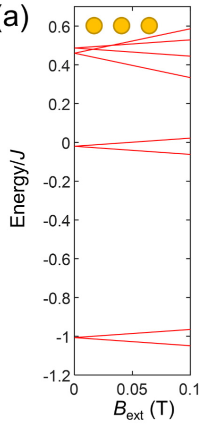

<table>
<tr><td>8</td><td>0.0043|↓↓↑⟩+0.0033|↓↑↓⟩+0.0625|↓↑↑⟩+0.0043|↑↓↓⟩+0.0810|↑↓↑⟩+0.0625|↑↑↓⟩+0.9928|↑↑↑⟩</td></tr>
<tr><td>7</td><td>-0.0053|↓↓↓⟩-0.0715|↓↓↑⟩-0.0630|↓↑↓⟩-0.5743|↓↑↑⟩-0.0715|↑↓↓⟩-0.5586|↑↓↑⟩-0.5743|↑↑↓⟩+0.1187|↑↑↑⟩</td></tr>
<tr><td>6</td><td>0.0934|↓↓↓⟩+0.5602|↓↓↑⟩+0.5911|↓↑↓⟩-0.0645|↓↑↑⟩+0.5602|↑↓↓⟩-0.0768|↑↓↑⟩-0.0645|↑↑↓⟩+0.0075|↑↑↑⟩</td></tr>
<tr><td>5</td><td>0.9954|↓↓↓⟩-0.0620|↓↓↑⟩-0.0387|↓↑↓⟩+0.0029|↓↑↑⟩-0.0620|↑↓↓⟩+0.0047|↑↓↑⟩+0.0029|↑↑↓⟩</td></tr>
<tr><td>4</td><td>-0.1047|↓↓↑⟩-0.6993|↓↑↑⟩+0.1047|↑↓↓⟩+0.6993|↑↑↓⟩</td></tr>
<tr><td>3</td><td>-0.6993|↓↓↑⟩+0.1047|↓↑↑⟩+0.6993|↑↓↓⟩-0.1047|↑↑↓⟩</td></tr>
<tr><td>2</td><td>0.0020|↓↓↓⟩+0.0247|↓↓↑⟩-0.0664|↓↑↓⟩+0.4010|↓↑↑⟩+0.0247|↑↓↓⟩-0.8200|↑↓↑⟩+0.4010|↑↑↓⟩+0.0165|↑↑↑⟩</td></tr>
<tr><td>1</td><td>0.0211|↓↓↓⟩+0.4203|↓↓↑⟩-0.8005|↓↑↓⟩-0.0356|↓↑↑⟩+0.4203|↑↓↓⟩+0.0553|↑↓↑⟩-0.0356|↑↑↓⟩-0.0010|↑↑↑⟩</td></tr>
</table>

(b)

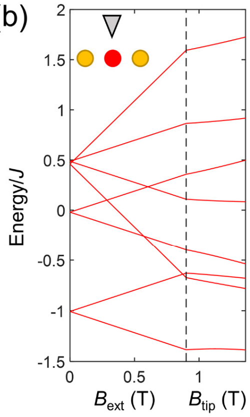

<table>
<tr><td>8</td><td>-0.0030|↓↓↑⟩-0.0012|↓↑↓⟩-0.0398|↓↑↑⟩-0.0030|↑↓↓⟩-0.0985|↑↓↑⟩-0.0398|↑↑↓⟩-0.9935|↑↑↑⟩</td></tr>
<tr><td>7</td><td>-0.0045|↓↓↓⟩-0.0821|↓↓↑⟩-0.0493|↓↑↓⟩-0.5997|↓↑↑⟩-0.0821|↑↓↓⟩-0.5051|↑↓↑⟩-0.5997|↑↑↓⟩+0.0986|↑↑↑⟩</td></tr>
<tr><td>6</td><td>0.1068|↓↓↑⟩+0.6990|↓↑↑⟩-0.1068|↑↓↓⟩-0.6990|↑↑↓⟩</td></tr>
<tr><td>5</td><td>0.1139|↓↓↓⟩+0.5256|↓↓↑⟩+0.6434|↓↑↓⟩-0.0436|↓↑↑⟩+0.5256|↑↓↓⟩-0.1288|↑↓↑⟩-0.0436|↑↑↓⟩+0.0123|↑↑↑⟩</td></tr>
<tr><td>4</td><td>0.6990|↓↓↑⟩-0.1068|↓↑↑⟩-0.6990|↑↓↓⟩+0.1068|↑↑↓⟩</td></tr>
<tr><td>3</td><td>-0.1628|↓↓↓⟩+0.0158|↓↓↑⟩-0.1166|↓↑↓⟩+0.3595|↓↑↑⟩+0.0158|↑↓↓⟩-0.8354|↑↓↑⟩+0.3595|↑↑↓⟩+0.0541|↑↑↑⟩</td></tr>
<tr><td>2</td><td>0.9603|↓↓↓⟩-0.1500|↓↓↑⟩+0.0573|↓↑↓⟩+0.0731|↓↑↑⟩-0.1500|↑↓↓⟩-0.1373|↑↓↑⟩+0.0731|↑↑↓⟩+0.0085|↑↑↑⟩</td></tr>
<tr><td>1</td><td>0.1959|↓↓↓⟩+0.4407|↓↓↑⟩-0.7529|↓↑↓⟩-0.0480|↓↑↑⟩+0.4407|↑↓↓⟩+0.0421|↑↓↑⟩-0.0480|↑↑↓⟩-0.0021|↑↑↑⟩</td></tr>
</table>

Supplementary Fig. 3. Energy level diagrams of 3-spin chain including weak anisotropy (J = 30 GHz and $J_{\mathrm{ani}} = -0.02\,J$). (a) At different B_{ext} (≤ 0.1 T). (b) At different B_{ext} (≤ 0.9 T) and B_{tip} (≤ 0.46 T, applied on spin $\mathbf{S}_2$). The

energy eigenstates (labeled from 1 to 8 in order of energy) are tabulated at the right panels for (a) $B_{\mathrm{ext}} = 0.1\ \mathrm{T}, B_{\mathrm{tip}} = 0$ and (b) $B_{\mathrm{ext}} = 0.9\ \mathrm{T}, B_{\mathrm{tip}} = 0.46\ \mathrm{T}$. Note that increasing the tip field causes the state mixing between different states, and (S_{T}, M) are no longer good quantum numbers.

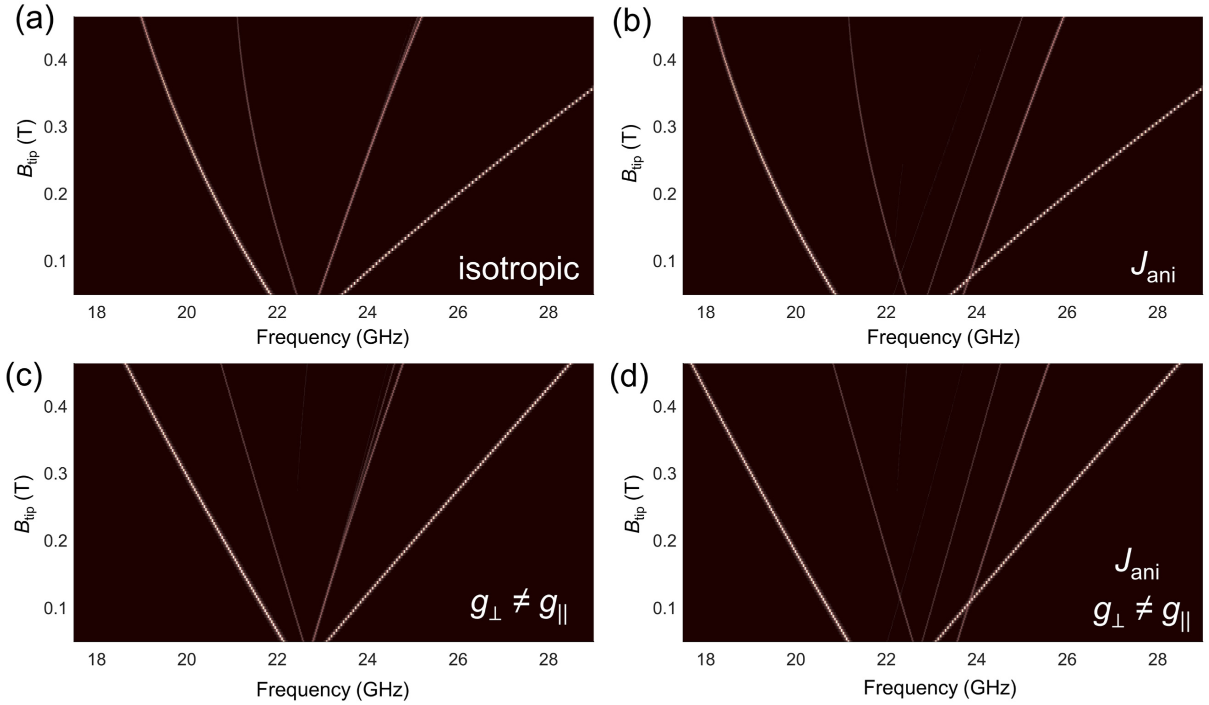

Supplementary Fig. 4. Simulated ESR spectra for J = 30 GHz at different B_{tip} ($B_{\mathrm{ext}} = 0.9$ T), using a tip field angle of 52.9° and (a) without J-anisotropy or g-anisotropy ($J_{\mathrm{ani}} = 0$, $g_\perp = g_\parallel = 1.8$); (b) with J-anisotropy ($J_{\mathrm{ani}} = -0.02\ J$); (c) with g-anisotropy ($J_{\mathrm{ani}} = 0$, $g_\perp = 0.6$, $g_\parallel = 1.8$); (d) with J-anisotropy and g-anisotropy ($J_{\mathrm{ani}} = -0.02\ J$, $g_\perp = 0.6$, $g_\parallel = 1.8$).

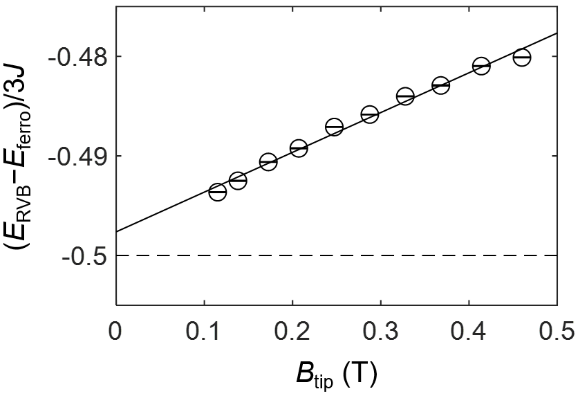

Supplementary Fig. 5. Energy difference between the RVB ground state and ferromagnetically aligned state ↑↓↓ + ↓↑↓ + ↓↓↑ at different B_{tip}. Solid line is a linear fit. Dashed line shows the calculated value at $B_{\mathrm{tip}} = 0$. Error bars are from the fitting uncertainties of the ESR frequencies with a 95% confidence.

Supplementary Note 6. 4-spin chain (J = 65 GHz)

6.1 dI/dV spectra

Tunneling spectra (dI/dV) are commonly used to observe spin excitations using the STM. Since tunneling spectra are thermally broadened, they are not able to resolve different transitions that can be resolved by ESR. Instead, tunneling spectra of the 4-spin chain at 1.2 K show only a single broad conductance step (Supplementary Fig. 6).

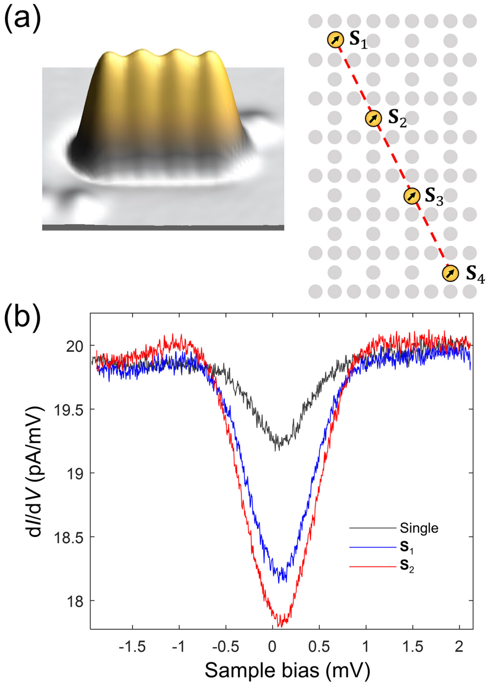

Supplementary Fig. 6. (a) STM image of the 4-spin chain (left) and the schematic of the adsorption sites of the four Ti_B atoms (right). (b) dI/dV spectra measured with a non-magnetic tip at different atomic spins $\mathbf{S}_1$ (blue) and $\mathbf{S}_2$ (red), as well as on a single Ti_B atom (black) (setpoint: V_{DC} = 10 mV, I_{DC} = 200 pA; B_{ext} = 0.9 T, T = 1.2 K).

6.2 Energy eigenstates and ESR simulations

The 4-spin chain at zero tip magnetic field has mirror symmetry, and the its eigenstates are thus labeled by the quantum number $\nu = \pm 1$ (Supplementary Fig. 7), which gives the phase of the wavefunction under the reflection operator. The presence of the tip magnetic field breaks the mirror symmetry, and we tabulated the eigenstates in Supplementary Fig. 8.

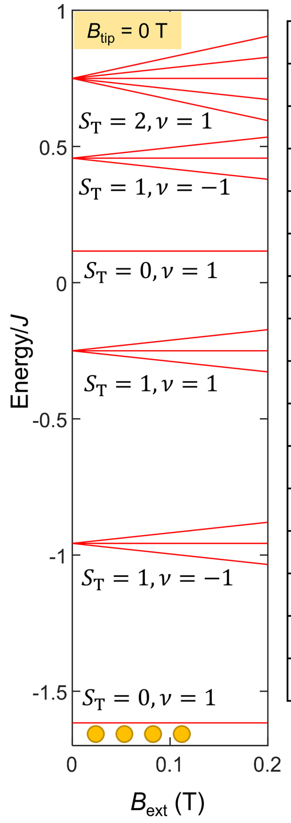

<table>
<tr><td>16</td><td>|↑↑↑↑⟩</td></tr>
<tr><td>15</td><td>1/2*(|↑↑↑↓⟩+|↑↑↓↑⟩+|↑↓↑↑⟩+|↓↑↑↑⟩)</td></tr>
<tr><td>14</td><td>1/√6*(|↓↓↑↑⟩+|↓↑↓↑⟩+|↓↑↑↓⟩+|↑↓↓↑⟩+|↑↓↑↓⟩+|↑↑↓↓⟩)</td></tr>
<tr><td>13</td><td>1/2*(|↓↓↓↑⟩+|↓↓↑↓⟩+|↓↑↓↓⟩+|↑↓↓↓⟩)</td></tr>
<tr><td>12</td><td>|↓↓↓↓⟩</td></tr>
<tr><td>11</td><td>0.6533|↓↑↑↑⟩+0.2706|↑↓↑↑⟩-0.2706|↑↑↓↑⟩-0.6533|↑↑↑↓⟩</td></tr>
<tr><td>10</td><td>0.6533|↓↓↑↑⟩+0.2706|↓↑↓↑⟩-0.2706|↑↓↑↓⟩-0.6533|↑↑↓↓⟩</td></tr>
<tr><td>9</td><td>0.6533|↓↓↓↑⟩+0.2706|↓↓↑↓⟩-0.2706|↓↑↓↓⟩-0.6533|↑↓↓↓⟩</td></tr>
<tr><td>8</td><td>-0.5577|↓↓↑↑⟩+0.1494|↓↑↓↑⟩+0.4083|↓↑↑↓⟩+0.4083|↑↓↓↑⟩+0.1494|↑↓↑↓⟩-0.5577|↑↑↓↓⟩</td></tr>
<tr><td>7</td><td>1/2*(|↑↑↑↓⟩-|↑↑↓↑⟩-|↑↓↑↑⟩+|↓↑↑↑⟩)</td></tr>
<tr><td>6</td><td>1/√2*(|↓↑↑↓⟩-|↑↓↓↑⟩)</td></tr>
<tr><td>5</td><td>1/2*(|↓↓↓↑⟩-|↓↓↑↓⟩-|↓↑↓↓⟩+|↑↓↓↓⟩)</td></tr>
<tr><td>4</td><td>0.2706|↓↑↑↑⟩-0.6533|↑↓↑↑⟩+0.6533|↑↑↓↑⟩-0.2706|↑↑↑↓⟩</td></tr>
<tr><td>3</td><td>-0.2706|↓↓↑↑⟩+0.6533|↓↑↓↑⟩-0.6533|↑↓↑↓⟩+0.2706|↑↑↓↓⟩</td></tr>
<tr><td>2</td><td>-0.2706|↓↓↓↑⟩+0.6533|↓↓↑↓⟩-0.6533|↓↑↓↓⟩+0.2706|↑↓↓↓⟩</td></tr>
<tr><td>1</td><td>0.1494|↓↓↑↑⟩-0.5577|↓↑↓↑⟩+0.4083|↓↑↑↓⟩+0.4083|↑↓↓↑⟩-0.5577|↑↓↑↓⟩+0.1494|↑↑↓↓⟩</td></tr>
</table>

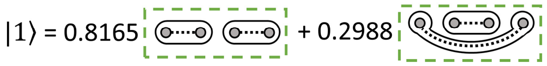

Supplementary Fig. 7. Energy level diagrams of 4-spin chain (J = 65 GHz) at different B_{ext} (≤ 0.2 T). The energy eigenstates (labeled from 1 to 16 in order of energy) are tabulated at the right panel for $B_{ext} = 0.2$ T, $B_{tip} = 0$.

(a)

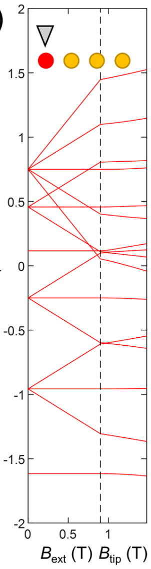

	|↓↓↓↓⟩	|↓↓↓↑⟩	|↓↓↑↓⟩	|↓↓↑↑⟩	|↓↑↓↓⟩	|↓↑↓↑⟩	|↓↑↑↓⟩	|↓↑↑↑⟩	|↑↓↓↓⟩	|↑↓↓↑⟩	|↑↓↑↓⟩	|↑↓↑↑⟩	|↑↑↓↓⟩	|↑↑↓↑⟩	|↑↑↑↓⟩	|↑↑↑↑⟩
16	0.0000	-0.0001	0.0000	-0.0043	0.0000	-0.0023	-0.0015	-0.1148	0.0000	-0.0009	-0.0006	-0.0515	-0.0003	-0.0244	-0.0143	-0.9916
15	-0.0001	-0.0043	-0.0038	-0.0821	-0.0025	-0.0748	-0.0734	-0.3678	-0.0011	-0.0428	-0.0383	-0.4686	-0.0234	-0.5371	-0.5719	0.0891
14	-0.0012	-0.0211	-0.0214	-0.0171	-0.0191	-0.0910	-0.1418	0.6543	-0.0114	-0.1128	-0.1309	0.3890	-0.1343	-0.1446	-0.5611	-0.0835
13	-0.0058	-0.0841	-0.0840	-0.2817	-0.0784	-0.3151	-0.3271	-0.1247	-0.0501	-0.4042	-0.4328	-0.0599	-0.5035	0.1037	0.2393	0.0147
12	-0.0360	-0.0811	-0.1296	0.6165	-0.1881	0.3484	0.1264	-0.1081	-0.1951	0.1512	-0.1375	0.0123	-0.5708	0.0057	-0.0709	0.0092
11	0.1409	0.3672	0.3898	0.1594	0.4580	0.0567	-0.0401	-0.0400	0.6052	0.0121	-0.1088	0.0126	-0.2700	0.0167	-0.0159	0.0031
10	-0.1151	0.3537	0.1786	-0.4085	-0.0768	0.0511	0.2194	0.3289	-0.2401	0.3361	0.1237	-0.2332	-0.3761	-0.2584	0.2216	-0.0218
9	-0.2234	0.4455	0.2416	-0.1371	-0.0670	-0.0403	-0.0979	-0.3952	-0.3457	0.0893	0.0156	0.3184	-0.0094	0.3921	-0.3450	0.0253
8	0.2989	-0.3532	-0.2218	-0.4623	0.0016	0.0758	0.2353	-0.1882	0.2766	0.3190	0.1468	0.1725	-0.2945	0.2365	-0.2326	0.0116
7	0.9063	0.2400	0.0642	0.0670	-0.1560	-0.0282	-0.0830	0.0091	-0.2713	-0.0248	-0.0215	-0.0103	0.0687	-0.0167	0.0223	-0.0005
6	0.0263	0.0463	0.1063	-0.0508	0.0289	0.0333	0.7352	-0.0421	-0.0728	-0.6464	0.0605	0.0664	-0.0567	-0.0057	-0.0675	0.0021
5	0.0023	0.0005	0.0104	0.0045	0.0137	0.1099	0.0043	0.3041	-0.0137	-0.0607	-0.0658	-0.6532	0.0367	0.6260	-0.2585	-0.0131
4	0.0630	-0.5174	0.4828	0.0050	0.5315	-0.0088	-0.0636	-0.0015	-0.4443	0.0565	0.0491	0.0036	-0.0749	-0.0051	0.0063	0.0001
3	-0.0065	-0.0255	0.0499	0.2666	-0.1246	-0.5934	-0.0658	0.0244	0.0681	-0.0542	0.6815	-0.0724	-0.2592	0.0903	-0.0242	-0.0009
2	0.0158	0.2649	-0.6454	0.0603	0.6369	-0.1851	0.0850	0.0026	-0.2397	0.0548	0.0082	-0.0097	-0.0361	0.0165	-0.0078	-0.0001
1	-0.0016	-0.0671	0.1293	0.1642	-0.0942	-0.5932	0.4170	0.0049	0.0295	0.3809	-0.5008	-0.0212	0.1326	0.0423	-0.0253	-0.0001

(b)

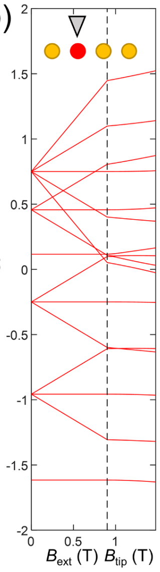

	|↓↓↓↓⟩	|↓↓↓↑⟩	|↓↓↑↓⟩	|↓↓↑↑⟩	|↓↑↓↓⟩	|↓↑↓↑⟩	|↓↑↑↓⟩	|↓↑↑↑⟩	|↑↓↓↓⟩	|↑↓↓↑⟩	|↑↓↑↓⟩	|↑↓↑↑⟩	|↑↑↓↓⟩	|↑↑↓↑⟩	|↑↑↑↓⟩	|↑↑↑↑⟩
16	0.0000	0.0001	0.0001	0.0043	0.0000	0.0020	0.0012	0.0542	0.0001	0.0032	0.0021	0.0920	0.0009	0.0436	0.0256	0.9930
15	-0.0002	-0.0051	-0.0042	-0.0701	-0.0023	-0.0508	-0.0437	-0.4690	-0.0036	-0.0706	-0.0642	-0.4330	-0.0394	-0.5101	-0.5491	0.1031
14	0.0004	0.0018	0.0041	-0.0920	0.0044	-0.0021	0.0361	-0.7014	0.0082	0.0491	0.0775	-0.2744	0.0710	0.2327	0.5948	0.0381
13	-0.0069	-0.0795	-0.0756	-0.3472	-0.0596	-0.3991	-0.4272	0.0030	-0.0766	-0.3601	-0.3901	0.0624	-0.4595	0.0888	0.1066	-0.0073
12	0.0181	-0.0293	0.0218	-0.5533	0.0991	-0.3326	-0.1126	0.0725	0.1737	-0.0969	0.1814	0.0704	0.6876	-0.0094	-0.0589	-0.0060
11	0.1252	0.4439	0.4742	0.0132	0.5401	0.0064	-0.0103	-0.0083	0.4767	-0.0685	-0.1022	0.0026	-0.1663	0.0101	0.0137	-0.0002
10	-0.0267	0.1334	0.0760	-0.4181	-0.0537	0.0687	0.2615	-0.3005	-0.1460	0.2493	0.0994	0.3758	-0.2405	0.4049	-0.4214	-0.0248
9	0.0810	-0.2831	-0.1506	0.4632	0.1087	-0.0154	-0.3244	-0.3191	0.2745	-0.1127	-0.1443	0.2757	0.2244	0.3626	-0.3001	-0.0174
8	-0.4001	0.5802	0.2386	0.2290	-0.2066	-0.0549	-0.2298	-0.0815	-0.3998	-0.1810	-0.0859	0.0627	0.2745	0.0822	-0.0527	-0.0038
7	0.8998	0.2011	0.0601	0.0569	-0.1119	-0.0208	-0.0691	-0.0165	-0.3335	-0.0509	-0.0116	0.0127	0.1034	0.0152	-0.0095	-0.0007
6	-0.0012	0.0907	-0.0246	-0.0714	0.0033	0.0489	-0.6415	0.0753	0.0266	0.7387	0.0463	-0.1257	-0.0447	-0.0048	-0.0019	0.0063
5	-0.0091	-0.0657	0.0657	-0.0029	0.0608	-0.0436	0.0704	0.2707	-0.0734	-0.0624	-0.0279	-0.6891	0.0222	0.6048	-0.2355	0.0289
4	0.0720	0.4607	-0.4482	-0.0643	-0.5002	0.0982	0.0578	0.0383	0.5375	-0.0659	-0.0704	-0.0881	-0.0133	0.0782	-0.0253	0.0037
3	0.0076	0.0804	-0.0094	0.2927	-0.0808	-0.6690	0.0334	-0.0165	0.0780	0.0372	0.6184	0.0018	-0.2466	0.0130	-0.0377	-0.0001
2	0.0410	-0.2920	0.6811	0.0138	-0.6009	-0.0413	0.0139	-0.0015	0.2589	0.0770	-0.1115	0.0036	0.0200	-0.0082	0.0071	-0.0001
1	-0.0033	0.0649	-0.1001	0.1399	0.0593	-0.5059	0.3862	-0.0081	-0.0296	0.4212	-0.5945	0.0123	0.1540	-0.0327	0.0302	-0.0004

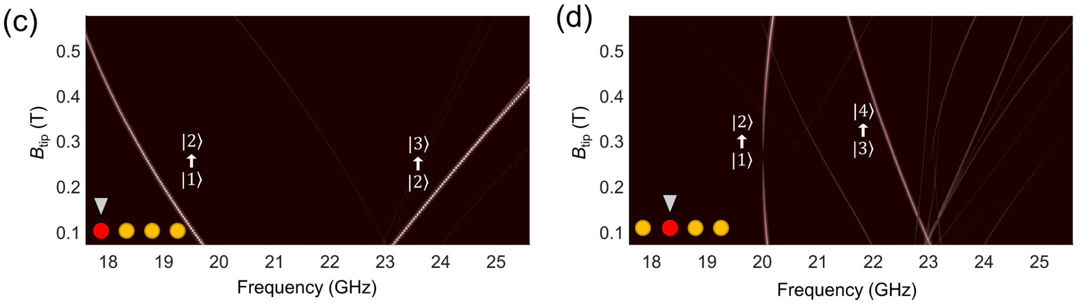

Supplementary Fig. 8. (a) Energy level diagrams of 4-spin chain (J = 65 GHz) at different B_{ext} (≤ 0.9 T) and B_{tip} (≤ 0.576 T, applied on spin $\mathbf{S}_1$). The energy eigenstates (labeled from 1 to 16 in order of energy) are tabulated at the right panel for $B_{ext} = 0.9$ T, $B_{tip} = 0.576$ T. (b) Same (a) except B_{tip} is applied on spin $\mathbf{S}_2$. (c, d) ESR simulations for B_{tip} applied on spin $\mathbf{S}_1$ or $\mathbf{S}_2$, using a tip field angle of 52.9°. Note that the |3⟩ → |4⟩ transition in (d) was not observed in the experiment.

6.3 ESR spectra with a different STM tip

The 4-spin chain responds differently using a STM tip having a moment that is strongly tilted with respect to z (Supplementary Fig. 9). More ESR transitions are detected, likely due to different ESR selection rule parameters. Note that for the tip used in Supplementary Fig. 9, not all ESR transitions in the simulated spectra match the experimental data and some ESR peak shifts to the different direction than the simulations. These discrepancies may result from the multiple spin centers on the tip[8]. The tip dependence of the ESR spectra suggest that different STM tips have different local magnetic configurations at the apex[8]. This presents an opportunity for future studies, but also constitutes a complication for interpreting the spectra and likely requires more elaborate models of the tip's magnetic structure.

The ESR spectra presented in the main text could be well-reproduced by simple modeling of the tip as a single spin center. In future studies, the STM tip should be firstly characterized by ESR measurement on smaller spin structures and make sure the complexity of the tip behavior will not obscure the intrinsic physics of interest.

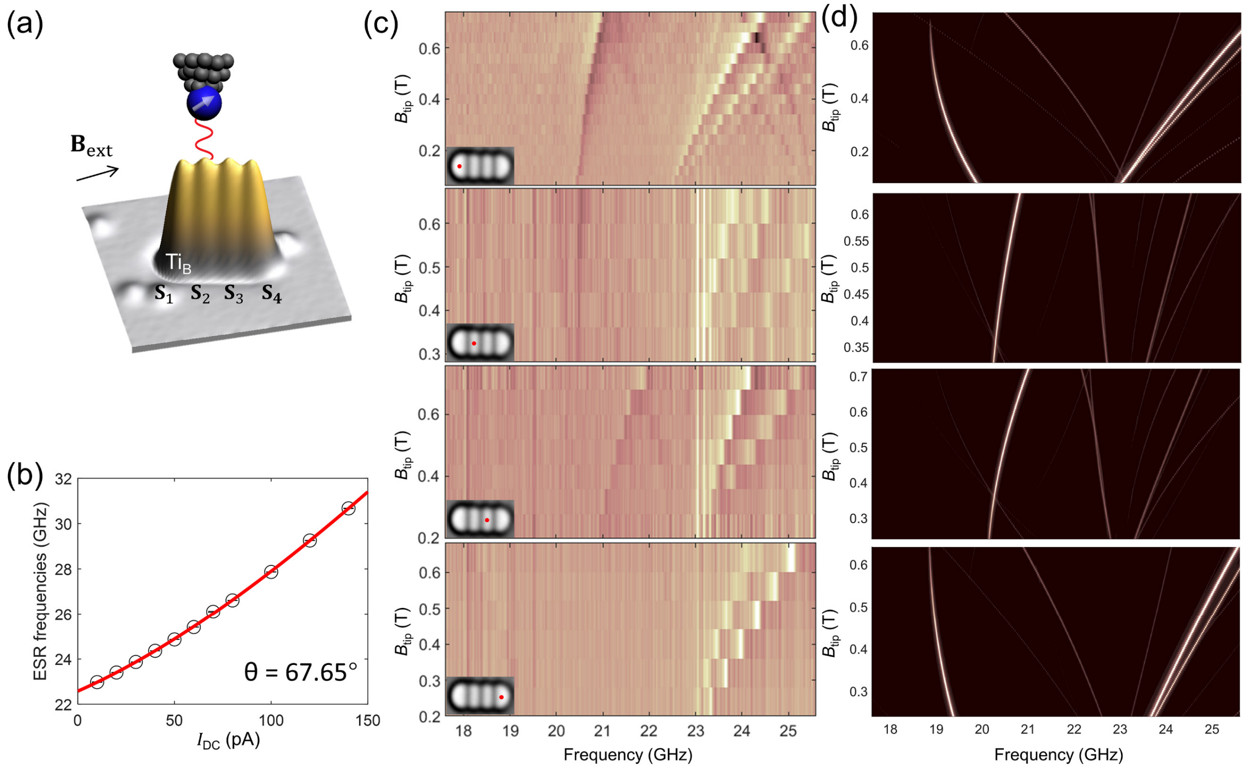

Supplementary Fig. 9. ESR of a 4-spin chain with a different STM tip than the tip used in Fig. 3 in the main text. (a) Experimental setup showing a 4-spin chain with a nearest-neighboring coupling of ~65 GHz. (b) Fitted ESR frequencies of a single Ti_B atom at different tip-Ti distances set by different I_{DC} at V_{DC} = 60 mV (I_{DC} = 20–180 pA, V_{RF} = 20–36 mV). Error bars are from the fitting uncertainties of the ESR frequencies with a 95% confidence. (c)

ESR spectra performed on each of the 4 atoms as a function of B_{tip} (setpoint: V_{DC} = 60 mV, I_{DC} = 10–140 pA, V_{RF} = 10–31 mV). The ESR spectra for this tip lack the mirror symmetry of the 4-spin chain, which is likely due to the blunt tip coupling to more than one atom during the measurement. (d) Simulations of the ESR spectra of the 4-spin chain, using a tip field angle of 67.65°.

Supplementary Note 7. Spin plaquette (*J* = 6 GHz)

The spin plaquette at zero tip magnetic field has translational symmetry, and the its eigenstates are thus labeled by the wavenumber k (Supplementary Fig. 10), which gives the phase of the wavefunction e^{ik} under the site-shift operator. The presence of the tip magnetic field breaks the translational symmetry, and we tabulated the eigenstates in Supplementary Fig. 11.

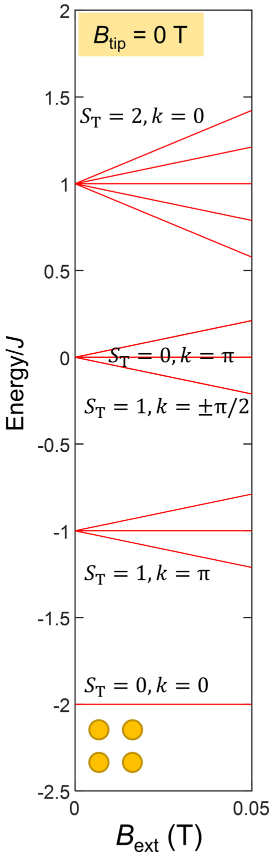

<table>
<tr><td>16</td><td>|↑↑↑↑⟩</td></tr>
<tr><td>15</td><td>1/2*(|↑↑↑↓⟩+|↑↑↓↑⟩+|↑↓↑↑⟩+|↓↑↑↑⟩)</td></tr>
<tr><td>14</td><td>1/√6*(|↓↓↑↑⟩+|↓↑↓↑⟩+|↓↑↑↓⟩+|↑↓↓↑⟩+|↑↓↑↓⟩+|↑↑↓↓⟩)</td></tr>
<tr><td>13</td><td>1/2*(|↓↓↓↑⟩+|↓↓↑↓⟩+|↓↑↓↓⟩+|↑↓↓↓⟩)</td></tr>
<tr><td>12</td><td>|↓↓↓↓⟩</td></tr>
<tr><td>11</td><td>1/2*(|↓↑↑↑⟩+ exp(+iπ/2)|↑↓↑↑⟩+ exp(+iπ)|↑↑↓↑⟩+ exp(+i3π/2)|↑↑↑↓⟩)</td></tr>
<tr><td>10</td><td>1/2*(|↓↑↑↑⟩+ exp(-iπ/2)|↑↓↑↑⟩+ exp(-iπ)|↑↑↓↑⟩+ exp(-i3π/2)|↑↑↑↓⟩)</td></tr>
<tr><td>9</td><td>1/2*(|↓↓↑↑⟩+ exp(+iπ/2)|↑↓↓↑⟩+ exp(+iπ)|↑↑↓↓⟩+ exp(+i3π/2)|↓↑↑↓⟩)</td></tr>
<tr><td>8</td><td>1/2*(|↓↓↑↑⟩-|↑↓↓↑⟩+|↑↑↓↓⟩-|↓↑↑↓⟩)</td></tr>
<tr><td>7</td><td>1/2*(|↓↓↑↑⟩+ exp(-iπ/2)|↑↓↓↑⟩+ exp(-iπ)|↑↑↓↓⟩+ exp(-i3π/2)|↓↑↑↓⟩)</td></tr>
<tr><td>6</td><td>1/2*(|↑↓↓↓⟩+ exp(+iπ/2)|↓↑↓↓⟩+ exp(+iπ)|↓↓↑↓⟩+ exp(+i3π/2)|↓↓↓↑⟩)</td></tr>
<tr><td>5</td><td>1/2*(|↑↓↓↓⟩+ exp(-iπ/2)|↓↑↓↓⟩+ exp(-iπ)|↓↓↑↓⟩+ exp(-i3π/2)|↓↓↓↑⟩)</td></tr>
<tr><td>4</td><td>1/2*(|↑↑↑↓⟩-|↑↑↓↑⟩+|↑↓↑↑⟩-|↓↑↑↑⟩)</td></tr>
<tr><td>3</td><td>1/√2*(|↓↑↓↑⟩-|↑↓↑↓⟩)</td></tr>
<tr><td>2</td><td>1/2*(|↓↓↓↑⟩-|↓↓↑↓⟩+|↓↑↓↓⟩-|↑↓↓↓⟩)</td></tr>
<tr><td>1</td><td>1/√12*(2|↓↑↓↑⟩+2|↑↓↑↓⟩-|↓↓↑↑⟩-|↓↑↑↓⟩-|↑↓↓↑⟩-|↑↑↓↓⟩)</td></tr>
</table>

Supplementary Fig. 10. Energy level diagrams of 4-spin plaquette (J = 6 GHz) at different B_{ext}. The energy eigenstates (labeled from 1 to 16 in order of energy) at $B_{\text{ext}} = 0.05$ T are tabulated in the right panel. Indices of degenerate states are indicated with the same background color. The wavenumber k gives the phase of the wavefunction under the site-shift operator. The RVB state (red) and the higher-energy singlet state (blue) are shown in color.

(a)

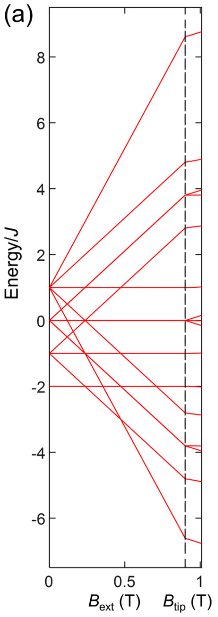

	|↓↓↓↓⟩	|↓↓↓↑⟩	|↓↓↑↓⟩	|↓↓↑↑⟩	|↓↑↓↓⟩	|↓↑↓↑⟩	|↓↑↑↓⟩	|↓↑↑↑⟩	|↑↓↓↓⟩	|↑↓↓↑⟩	|↑↓↑↓⟩	|↑↓↑↑⟩	|↑↑↓↓⟩	|↑↑↓↑⟩	|↑↑↑↓⟩	|↑↑↑↑⟩
16	0.0000	0.0000	0.0000	0.0001	0.0000	0.0000	0.0001	0.0352	0.0000	0.0000	0.0000	0.0037	0.0000	0.0008	0.0037	0.9994
15	0.0000	-0.0001	-0.0001	-0.0203	-0.0001	-0.0198	-0.0203	-0.4122	0.0000	-0.0025	-0.0040	-0.5136	-0.0025	-0.5486	-0.5136	0.0188
14	0.0000	0.0000	0.0000	0.0303	0.0000	0.0000	-0.0303	0.0000	0.0000	0.0000	0.0000	0.7065	0.0000	0.0000	-0.7065	0.0000
13	0.0000	0.0001	0.0000	-0.0015	0.0001	0.0258	-0.0015	-0.6825	0.0000	0.0036	0.0005	-0.1075	0.0036	0.7140	-0.1075	0.0243
12	0.0000	0.0000	0.0002	0.0267	0.0000	-0.0126	0.0267	-0.6025	0.0000	-0.0011	0.0069	0.4728	-0.0011	-0.4335	0.4728	0.0180
11	0.0001	0.0189	0.0192	0.3428	0.0189	0.3723	0.3428	0.0073	0.0040	0.4667	0.4344	-0.0191	0.4667	-0.0158	-0.0191	-0.0002
10	0.0000	-0.0303	0.0000	0.0000	0.0303	0.0000	0.0000	0.0000	0.0000	-0.7065	0.0000	0.0000	0.7065	0.0000	0.0000	0.0000
9	0.0002	0.0214	0.0015	-0.5321	0.0214	-0.0657	-0.5321	-0.0068	0.0059	0.4580	-0.0878	0.0249	0.4580	-0.0034	0.0249	0.0001
8	0.0000	0.0000	0.0000	-0.7065	0.0000	0.0000	0.7065	0.0000	0.0000	0.0000	0.0000	0.0303	0.0000	0.0000	-0.0303	0.0000
7	0.0001	0.0054	0.0464	-0.0621	0.0054	-0.6479	-0.0621	0.0000	0.0020	-0.0467	0.7520	-0.0001	-0.0467	0.0231	-0.0001	0.0000
6	0.0004	0.0177	-0.0349	0.3072	0.0177	-0.6602	0.3072	0.0019	0.0108	0.2629	-0.4842	-0.0110	0.2629	0.0213	-0.0110	0.0000
5	0.0273	0.4725	0.4310	0.0023	0.4725	0.0135	0.0023	0.0000	0.6040	-0.0255	-0.0262	0.0000	-0.0255	-0.0003	0.0000	0.0000
4	0.0407	-0.1104	-0.7126	-0.0066	-0.1104	0.0029	-0.0066	0.0000	0.6812	-0.0011	0.0450	0.0002	-0.0011	-0.0001	0.0002	0.0000
3	0.0000	0.7065	0.0000	0.0000	-0.7065	0.0000	0.0000	0.0000	0.0000	-0.0303	0.0000	0.0000	0.0303	0.0000	0.0000	0.0000
2	0.0386	-0.5131	0.5502	0.0039	-0.5131	-0.0068	0.0039	0.0000	0.4091	0.0216	-0.0303	-0.0001	0.0216	0.0001	-0.0001	0.0000
1	0.9981	0.0114	-0.0041	0.0000	0.0114	0.0001	0.0000	0.0000	-0.0601	-0.0003	0.0002	0.0000	-0.0003	0.0000	0.0000	0.0000

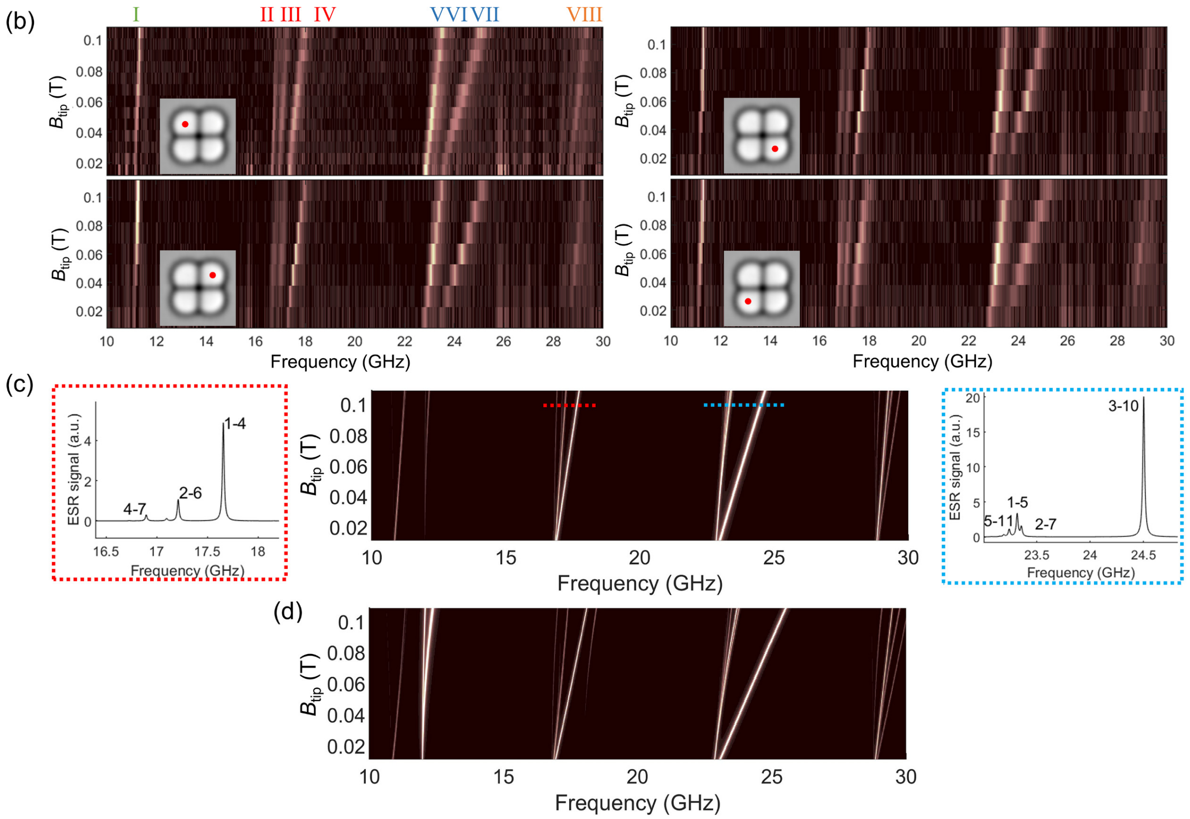

Supplementary Fig. 11. (a) Energy level diagrams of 4-spin plaquette (J = 6 GHz) at different B_{ext} (≤ 0.9 T) and B_{tip} (≤ 0.109 T, applied on spin $\mathbf{S}_1$). The energy eigenstates (labeled from 1 to 16 in order of energy) are tabulated at the right panel for $B_{ext} = 0.9$ T, $B_{tip} = 0.109$ T. (b) ESR spectra as a function of B_{tip} measured on four different spins (setpoint: V_{DC} = 50 mV, I_{DC} = 50–350 pA, V_{RF} = 3–14 mV). (c) Simulated ESR spectra using g = 1.81, J = 6 GHz, and a tip field angle of 50°, which yields better agreement with measured ESR spectra than using smaller angles, e.g. (d) a tip field angle of 10°. Left and right panels show the simulated ESR spectra corresponding to the red and blue dashed lines, with transitions labelled according to the initial and final states.

Supplementary Note 8. Spin plaquette (*J* = 25 GHz)

For the spin plaquette having strong coupling (*J* = 25 GHz) so the RVB state is the ground state, at least two transitions are prominently visible (Supplementary Fig. 12). Transition I ($|2\rangle \rightarrow |3\rangle$) accesses the Neel-like state $|3\rangle \approx |\uparrow\downarrow\uparrow\downarrow\rangle - |\downarrow\uparrow\downarrow\uparrow\rangle$, while transition II ($|4\rangle \rightarrow |10\rangle$) accesses a higher energy multiplet.

The ESR transitions indicated by the red arrows in Supplementary Fig. 12b resemble the transitions from the RVB ground state to the first excite state, as suggested by comparing with the simulations in Supplementary Fig. 12c. However, these transitions did not shift to higher frequencies at lower external field (Supplementary Fig. 12d), as they do in the simulations (Supplementary Fig. 12e). Therefore, they are more likely artifacts of transitions I and II, due to a transiently occupied reversal of the tip magnetic moment, as observed in fig. S7 of Ref. 8.

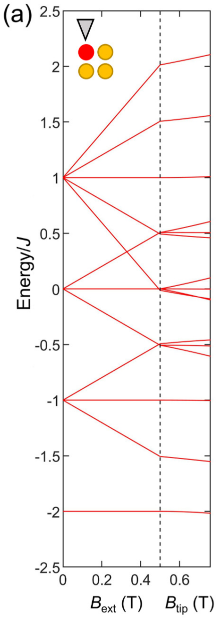

	|↓↓↓↓⟩	|↓↓↓↑⟩	|↓↓↑↓⟩	|↓↓↑↑⟩	|↓↑↓↓⟩	|↓↑↓↑⟩	|↓↑↑↓⟩	|↓↑↑↑⟩	|↑↓↓↓⟩	|↑↓↓↑⟩	|↑↓↑↓⟩	|↑↓↑↑⟩	|↑↑↓↓⟩	|↑↑↓↑⟩	|↑↑↑↓⟩	|↑↑↑↑⟩
16	0.0000	0.0000	0.0000	0.0021	0.0000	0.0016	0.0021	0.0798	0.0000	0.0006	0.0009	0.0339	0.0006	0.0225	0.0339	0.9954
15	-0.0001	-0.0024	-0.0028	-0.0584	-0.0024	-0.0539	-0.0584	-0.4373	-0.0012	-0.0307	-0.0361	-0.5052	-0.0307	-0.5280	-0.5052	0.0818
14	0.0039	0.0616	0.0650	0.3620	0.0616	0.3818	0.3620	-0.0857	0.0433	0.4406	0.4204	-0.0511	0.4406	-0.0301	-0.0511	0.0079
13	0.0000	0.0000	0.0000	0.1022	0.0000	0.0000	-0.1022	0.0000	0.0000	0.0000	0.0000	0.6997	0.0000	0.0000	-0.6997	0.0000
12	0.0068	0.0370	0.0364	0.0187	0.0370	-0.0204	0.0187	0.6933	0.0406	0.0202	0.0300	0.0626	0.0202	-0.7078	0.0626	-0.0439
11	0.1001	0.4765	0.4499	-0.0936	0.4765	-0.0612	-0.0936	-0.0406	0.5524	-0.0070	-0.0402	-0.0018	-0.0070	0.0600	-0.0018	0.0025
10	0.0000	-0.1022	0.0000	0.0000	0.1022	0.0000	0.0000	0.0000	0.0000	-0.6997	0.0000	0.0000	0.6997	0.0000	0.0000	0.0000
9	0.0791	0.0235	0.0480	0.5110	0.0235	0.0298	0.5110	-0.0811	0.0750	-0.4725	0.0476	0.0340	-0.4725	-0.0624	0.0340	0.0039
8	0.9772	-0.0757	-0.1530	-0.0316	-0.0757	0.0092	-0.0316	0.0074	0.0690	0.0403	-0.0081	-0.0037	0.0403	0.0041	-0.0037	-0.0003
7	0.0000	0.0000	0.0000	-0.6997	0.0000	0.0000	0.6997	0.0000	0.0000	0.0000	0.0000	0.1022	0.0000	0.0000	-0.1022	0.0000
6	-0.0053	-0.0002	-0.0080	0.0514	-0.0002	0.0682	0.0514	0.5529	0.0190	-0.0546	-0.0088	-0.4849	-0.0546	0.4551	-0.4849	-0.0218
5	0.1664	0.0460	0.6969	-0.0166	0.0460	-0.0639	-0.0166	0.0116	-0.6854	-0.0215	0.0803	-0.0108	-0.0215	0.0156	-0.0108	-0.0004
4	0.0000	0.6997	0.0000	0.0000	-0.6997	0.0000	0.0000	0.0000	0.0000	-0.1022	0.0000	0.0000	0.1022	0.0000	0.0000	0.0000
3	0.0107	0.0548	0.0459	0.0341	0.0548	0.6642	0.0341	-0.0133	-0.0962	0.0255	-0.7307	0.0189	0.0255	-0.0539	0.0189	0.0004
2	-0.0310	-0.5026	0.5216	-0.0478	-0.5026	0.1288	-0.0478	-0.0024	0.4472	-0.0116	0.0107	0.0047	-0.0116	-0.0083	0.0047	0.0001
1	0.0020	0.0609	-0.0800	-0.2965	0.0609	0.6162	-0.2965	-0.0078	-0.0383	-0.2747	0.5259	0.0189	-0.2747	-0.0311	0.0189	0.0002

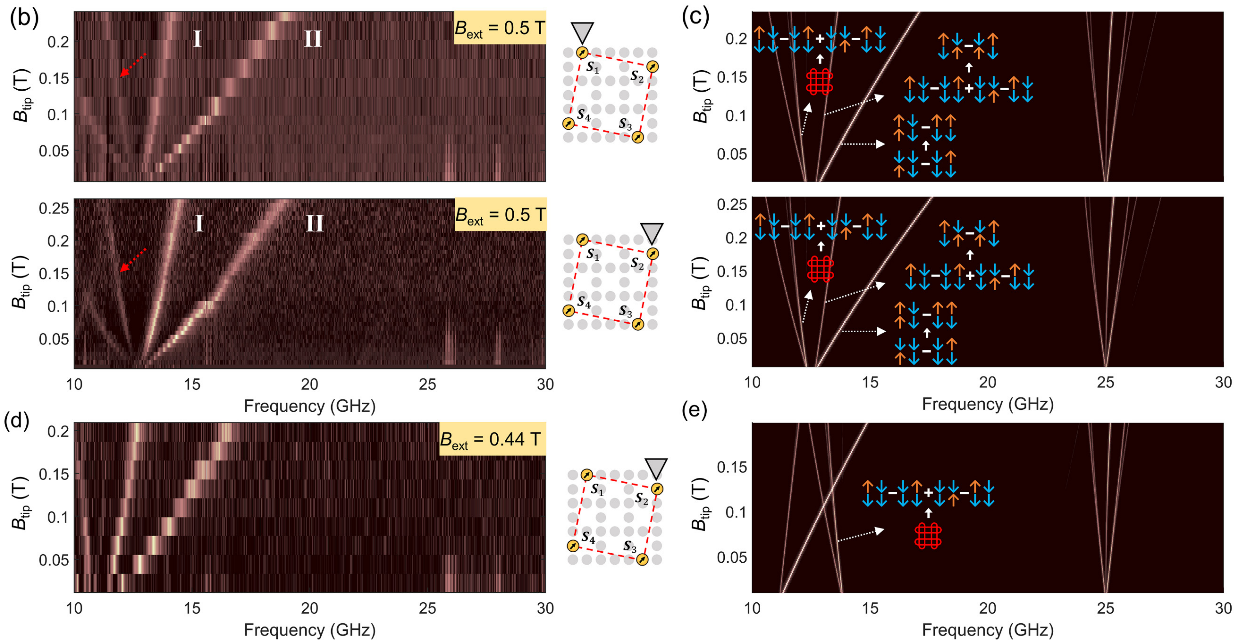

Supplementary Fig. 12. (a) Energy level diagram of 4-spin plaquette (J = 25 GHz) at different B_{ext} (≤ 0.5 T) and B_{tip} (≤ 0.26 T, applied on spin $\mathbf{S}_1$). The energy eigenstates (labeled from 1 to 16 in order of energy) are tabulated at the right panel for $B_{\mathrm{ext}} = 0.5$ T, $B_{\mathrm{tip}} = 0.26$ T. (b) ESR spectra as a function of B_{tip} measured on $\mathbf{S}_1$ and $\mathbf{S}_2$ (setpoint: V_{DC} = 50 mV, I_{DC} = 25–1000 pA, V_{RF} = 2–25 mV. $B_{\mathrm{ext}} = 0.5$ T). (c) Simulations of the ESR spectra at $B_{\mathrm{ext}} = 0.5$ T, using a tip field angle of 50°. (d, e) ESR spectra and simulations at $B_{\mathrm{ext}} = 0.44$ T, using a tip field angle of 50°.

Supplementary Note 9. Spin triangle

To explore the effect of classical spin frustration that comes from odd-length cycles we assembled a 3-spin triangle (Supplementary Fig. 13). For this 3-spin triangle, the coupling is less than 1 GHz, and the fully polarized Zeeman product state ↓↓↓ is the ground state for B_{ext} = 0.9 T studied here (see also Supplementary Fig. 13 for the eigenstates). Frustration increases as J_{31} is increased starting from zero. This frustration is evident as a reduction of the splitting between the two low-energy doublets, which should become degenerate for an ideal equilateral triangle $J_{12} = J_{23} = J_{31}$. Here the splitting is given by the difference between $|1\rangle \rightarrow |3\rangle$ and $|2\rangle \rightarrow |3\rangle$, which is ~0.4 GHz. The competition of spin frustration and local tip field can be directly visualized in the evolution of the ESR spectra with tip field. Increasing the tip field reduces the spin frustration by local spin polarization of the spin under the tip.

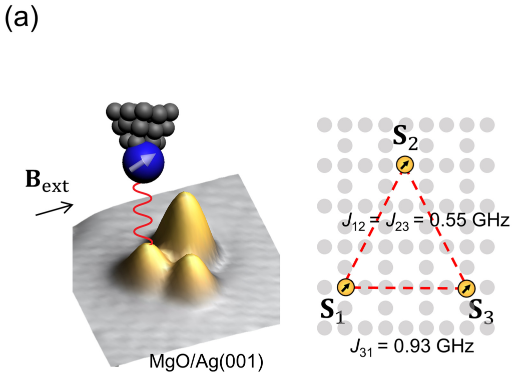

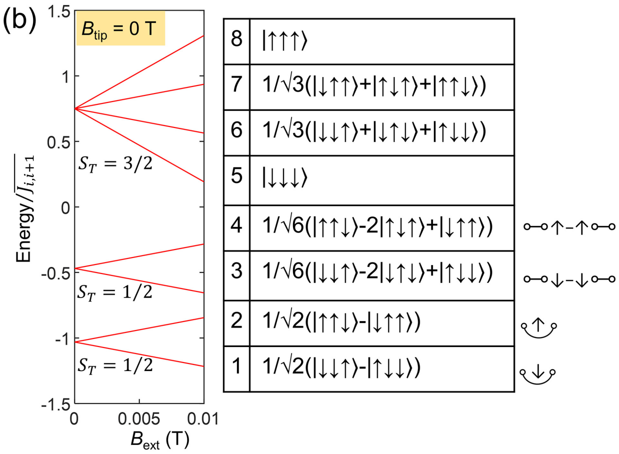

<table>
<tr><td>8</td><td>|↑↑↑⟩</td></tr>
<tr><td>7</td><td>1/√3(|↓↑↑⟩+|↑↓↑⟩+|↑↑↓⟩)</td></tr>
<tr><td>6</td><td>1/√3(|↓↓↑⟩+|↓↑↓⟩+|↑↓↓⟩)</td></tr>
<tr><td>5</td><td>|↓↓↓⟩</td></tr>
<tr><td>4</td><td>1/√6(|↑↑↓⟩-2|↑↓↑⟩+|↓↑↑⟩)</td></tr>
<tr><td>3</td><td>1/√6(|↓↓↑⟩-2|↓↑↓⟩+|↑↓↓⟩)</td></tr>
<tr><td>2</td><td>1/√2(|↑↑↓⟩-|↓↑↑⟩)</td></tr>
<tr><td>1</td><td>1/√2(|↓↓↑⟩-|↑↓↓⟩)</td></tr>
</table>

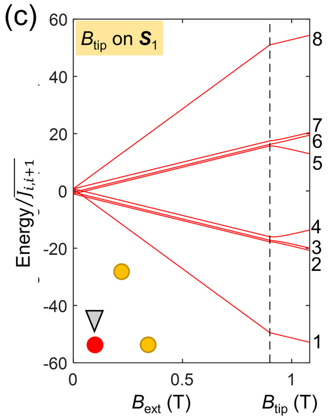

<table>
<tr><td>8</td><td>-0.0143|↓↑↑⟩-0.9999|↑↑↑⟩</td></tr>
<tr><td>7</td><td>0.0115|↓↓↑⟩+0.0089|↓↑↓⟩+0.1002|↓↑↑⟩+0.7895|↑↓↑⟩+0.6053|↑↑↓⟩-0.0017|↑↑↑⟩</td></tr>
<tr><td>6</td><td>0.0089|↓↓↑⟩-0.0117|↓↑↓⟩-0.0457|↓↑↑⟩+0.6114|↑↓↑⟩-0.7899|↑↑↓⟩</td></tr>
<tr><td>5</td><td>0.0017|↓↑↓⟩-0.9938|↓↑↑⟩+0.0515|↑↓↑⟩+0.0974|↑↑↓⟩+0.0142|↑↑↑⟩</td></tr>
<tr><td>4</td><td>0.0149|↓↓↓⟩+0.1042|↓↓↑⟩+0.0682|↓↑↓⟩+0.9921|↑↓↓⟩-0.0018|↑↓↑⟩-0.0012|↑↑↓⟩</td></tr>
<tr><td>3</td><td>0.0020|↓↓↓⟩-0.5372|↓↓↑⟩-0.8356|↓↑↓⟩+0.1139|↑↓↓⟩+0.0078|↑↓↑⟩+0.0123|↑↑↓⟩</td></tr>
<tr><td>2</td><td>0.8369|↓↓↑⟩-0.5449|↓↑↓⟩-0.0504|↑↓↓⟩-0.0122|↑↓↑⟩+0.0081|↑↑↓⟩</td></tr>
<tr><td>1</td><td>0.9999|↓↓↓⟩−0.0151|↑↓↓⟩</td></tr>
</table>

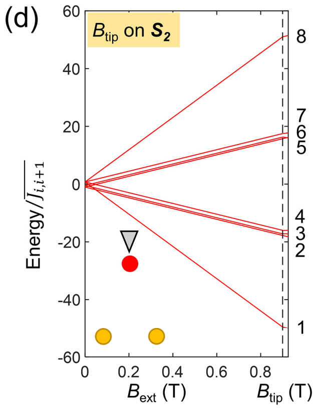

<table>
<tr><td>8</td><td>-0.0023|↑↓↑⟩-|↑↑↑⟩</td></tr>
<tr><td>7</td><td>-0.0016|↓↓↑⟩-0.6632|↓↑↑⟩-0.0016|↑↓↓⟩-0.3469|↑↓↑⟩-0.6632|↑↑↓⟩</td></tr>
<tr><td>6</td><td>-0.0017|↓↓↑⟩-0.7071|↓↑↑⟩+0.0017|↑↓↓⟩+0.7071|↑↑↓⟩</td></tr>
<tr><td>5</td><td>0.2453|↓↑↑⟩-0.9379|↑↓↑⟩+0.2453|↑↑↓⟩+0.0022|↑↑↑⟩</td></tr>
<tr><td>4</td><td>0.0020|↓↓↓⟩+0.3815|↓↓↑⟩+0.8420|↓↑↓⟩+0.3815|↑↓↓⟩</td></tr>
<tr><td>3</td><td>-0.0014|↓↓↓⟩+0.5954|↓↓↑⟩-0.5395|↓↑↓⟩-0.0014|↓↑↑⟩+0.5954|↑↓↓⟩-0.0014|↑↑↓⟩</td></tr>
<tr><td>2</td><td>-0.7071|↓↓↑⟩+0.0017|↓↑↑⟩+0.7071|↑↓↓⟩-0.0017|↑↑↓⟩</td></tr>
<tr><td>1</td><td>|↓↓↓⟩-0.0025|↓↑↓⟩</td></tr>
</table>

Supplementary Fig. 13. (a) Experimental setup showing a 3-spin triangle with couplings of $J_{12} = J_{23} = 0.55$ GHz and $J_{31} = 0.93$ GHz. Right: Adsorption sites of the three Ti atoms. (b) Energy level diagrams at different B_{ext} ($\leq$ 0.01 T). Energy eigenstates (labeled from 1 to 8 in order of energy) are tabulated at the right panels for $B_{\text{ext}} =$ 0.01 T. (c) Energy level diagrams at different B_{ext} ($\leq$ 0.9 T) and B_{tip} ($\leq$ 0.183 T, applied on spin $\mathbf{S}_1$). Right shows the energy eigenstates at $B_{\text{ext}} = 0.9$ T, $B_{\text{tip}} = 0.183$ T. (d) Same as (c) except $B_{\text{tip}} \leq$ 0.026 T, applied on $\mathbf{S}_2$.

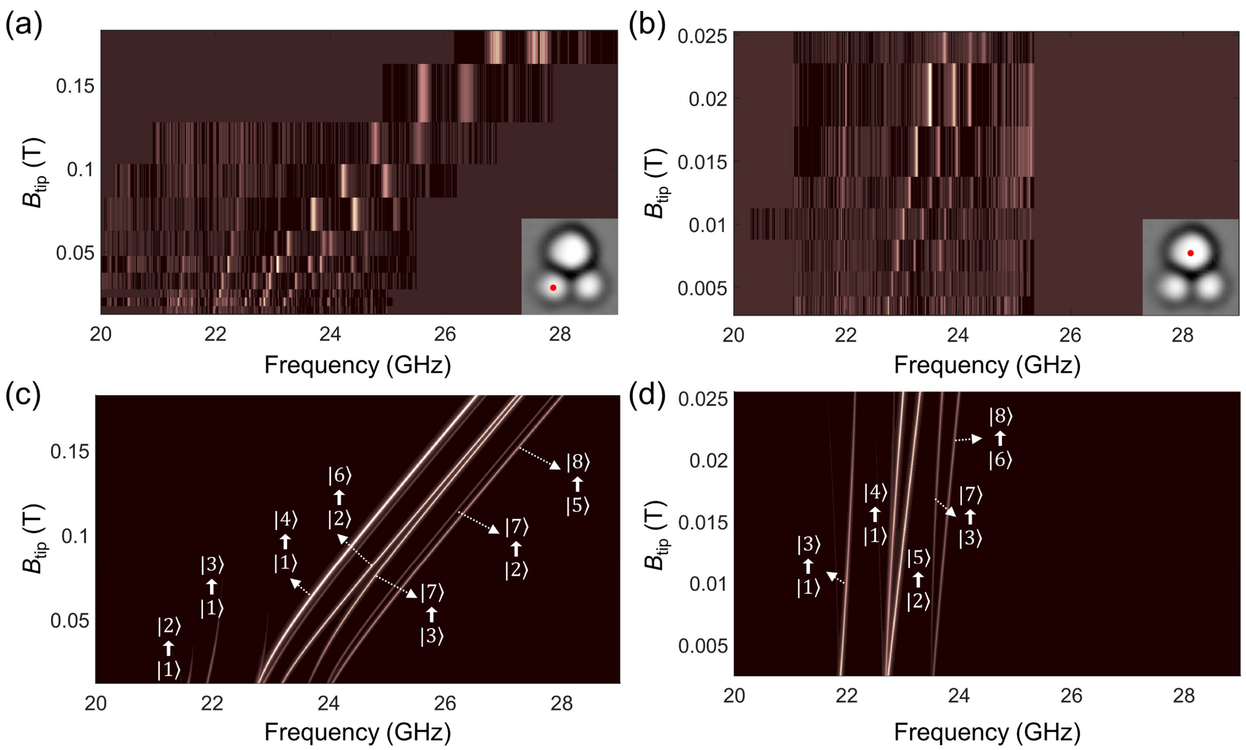

Supplementary Fig. 14. (a) ESR spectra at different B_{tip} measured on the left Ti_O molecule (setpoint: V_{DC} = 40–60 mV, I_{DC} = 1.7–18 pA, V_{RF} = 8–35 mV) and (b) on the Ti_B molecule (setpoint: V_{DC} = 40, 80 mV, I_{DC} = 6–25 pA, V_{RF} = 20–40 mV). B_{ext} = 0.9 T, T = 1.2 K. (c, d) Simulations of the ESR spectra, using a tip field angle of 10°.

Supplementary Note 10. References

1 K. Yang *et al.* Engineering the eigenstates of coupled spin-1/2 atoms on a surface. *Phys. Rev. Lett.* **119**, 227206 (2017).

2 Y. Bae *et al.* Enhanced quantum coherence in exchange coupled spins via singlet-triplet transitions. *Sci. Adv.* **4**, eaau4159 (2018).

3 K. Yang *et al.* Tuning the exchange bias on a single atom from 1 mT to 10 T. *Phys. Rev. Lett.* **122**, 227203 (2019).

4 K. Yang *et al.* Electrically controlled nuclear polarization of individual atoms. *Nat. Nanotechnol.* **13**, 1120-1125 (2018).

5 W. K. Wootters. Entanglement of formation of an arbitrary state of two qubits. *Phys. Rev. Lett.* **80**, 2245-2248 (1998).

6 D. Gatteschi, R. Sessoli & J. Villain. *Molecular nanomagnets*. (Oxford Univ. Press, 2006).

7 M. Steinbrecher *et al.* Quantifying the interplay between fine structure and geometry of an individual molecule on a surface. arXiv:2007.01928 (2020).

8 P. Willke *et al.* Magnetic resonance imaging of single atoms on a surface. *Nat. Phys.* **15**, 1005-1010 (2019).
