# Supplementary material for: Probing resonating valence bond states in artificial quantum magnets
Source: Nat Commun. 2021 Feb 12;12:993. doi: 10.1038/s41467-021-21274-5 (PMC7881118; doi:10.1038/s41467-021-21274-5)
Supplement: Supplementary file 4 — Description of Additional Supplementary Files [file 41467_2021_21274_MOESM4_ESM.pdf]

Title: Supplementary Movie 1:
Description: A series of STM images showing the process of building the spin plaquette.

Title: Supplementary Movie 2:
Description: A series of STM images showing the process of building the 4-spin chain.
